# Mass Spectrometry Proteomics: A Key to Faster Drug Discovery

**DOI:** 10.1021/acs.jmedchem.5c01986

**Published:** 2025-12-23

**Authors:** Lorenzo Tagliazucchi, Maria Paola Costi

**Affiliations:** Department of Life Sciences, University of Modena and Reggio Emilia, Via G. Campi 103, 41125 Modena, Italy

## Abstract

Mass spectrometry (MS)-based proteomics is a disruptive platform in drug discovery that offers an exhaustive view of the proteome’s complexity. Focusing on bottom-up MS proteomics, this technology enables high-throughput analysis of protein expression, interactions, and modifications, far surpassing the capabilities of traditional single-protein methods. The MS proteomics toolbox is essential in both early- and late-stage development of new drugs. The techniques discussed here, such as unlabeled and labeled proteomics and chemoproteomic approaches (e.g., thermal proteome profiling and photoaffinity labeling), facilitate target binding site exploration and the identification of putative off-targets. By accelerating the identification of new druggable proteins and supporting early biomarker discovery, MS proteomics significantly accelerates the preclinical-to-clinical transition. Ongoing progress in data acquisition, new computational tools, and artificial intelligence further enhances the high-throughput properties of these approaches, marking a significant step toward personalized medicine.

## Graphical abstract

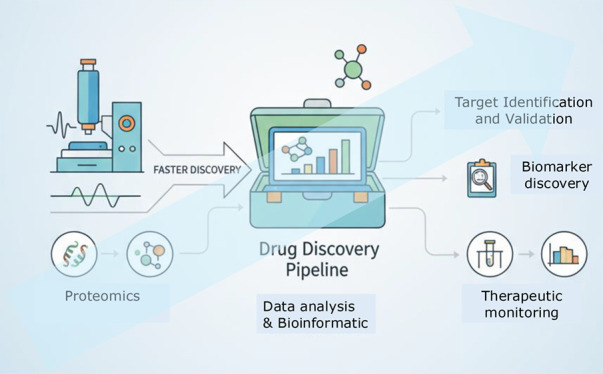

## 1 Introduction

Drug discovery remains a time-consuming and failure-prone process, which often requires over a decade and a consistent financial investment to bring a new therapeutic agent to the market.^1^ As a result, there is a growing need for more robust and integrative technologies to update drug discovery workflows and improve success rates.^2,3^ Mass spectrometry (MS)-based proteomics has emerged as an innovative approach in this context, as it offers a complete qualitative and quantitative insight into protein expression, post-translational modifications, and their interaction with other proteins or molecules.^4,5^ Unlike genomic or transcriptomic tools, which provide an indirect description of cellular function, MS proteomics directly measures the functional biomolecules that regulate nearly all biological processes.^6^ Despite its innovation potential, MS proteomics remains underexploited in medicinal chemistry, partly due to its technological complexity, data interpretation challenges, and a historical disconnection between proteomics and the drug design process.^7^ Thus, this review aims to provide a structured overview of how MS proteomics can be exploited across the drug discovery pipeline from early target identification to translational applications in precision medicine.

The journey begins with target discovery, where comparative MS-based proteomics between healthy and diseased states can reveal differentially expressed proteins and dysregulated pathways. Untargeted and targeted chemoproteomics strategies, such as thermal proteome profiling (TPP) and other chemical proteomics techniques, allow the identification of druggable proteins, even in complex samples^8,9^ (Figure 1, from left to right, drug discovery process and MS technologies progression). These techniques represent a critical starting point to identify the modifiable targets. Next, target engagement studies determine whether and how drug candidates interact with their targets in biological systems. Activity-based protein profiling (ABPP), photoaffinity labeling (PAL), limited proteolysis coupled to MS (LiP-MS), and cross-linking MS (XL-MS) are used to evaluate binding specificity, stoichiometry, and cellular accessibility.^5^ When combined with intact-cell approaches, real-time, in situ feedback on compound efficacy and selectivity information can be achieved. Once validated, the targets are subjected to biochemical mechanistic investigation. MS proteomic readouts can reveal changes in protein expression levels, protein complex formation, and pathway activation, all of which contribute to the drug’s mechanism of action (MoA).^10^ Covalent chemical proteomic methods, such as hydrogen-deuterium exchange (HDX-MS), help to map ligand-binding sites and identify conformational changes, providing medicinal chemists with data to guide structure-activity relationship (SAR) optimization.^11^ Fragment-based drug discovery (FBDD) can also be pursued by MS chemoproteomics, where weak but specific fragment-protein interactions are characterized and refined toward high-affinity leads.^12^ MS proteomics has the possibility to play a central role in understanding off-target effects and resistance mechanisms, which are key drivers of drug failure.^13^ Proteome-wide analysis can detect alterations in signaling pathways and protein abundance associated with adverse outcomes or compensatory responses.^14^ Techniques such as co-immunoprecipitation followed by MS (Co-IP-MS) allow for the identification of toxicity markers or resistance biomarkers early in development.^15^ This strategy supports the design of safer and more effective drugs and facilitates combination therapy strategies to prevent drug resistance onset, e.g., the synthetic lethality paradigm.^16^ In translational and clinical research, MS proteomics bridges preclinical insights with patient data, contributing to precision medicine.^17,18^ Clinical proteomics enables patient stratification by identifying disease-specific signatures, predictive biomarkers, and pharmacodynamic markers from biopsies, plasma, or other biofluids. Data-independent acquisition (DIA) methods and isobaric labeling (e.g., TMT or iTRAQ) have improved the reproducibility and throughput of proteomics in complex clinical matrices.^19^ These advances, coupled with the availability of large clinical metadata sets, support early detection of therapy resistance and real-time monitoring of therapeutic response.^20^ Recent innovations in instrumentation (e.g., hybrid MS with different fragmentations) and computational platforms (e.g., Skyline, Proteome Discoverer) have improved data depth and quality.^21^ Artificial intelligence (AI) now supports pattern recognition, predictive modeling, and automated hypothesis generation, which are crucial for navigating complex MS proteomic data.^22^

**Figure 1:**
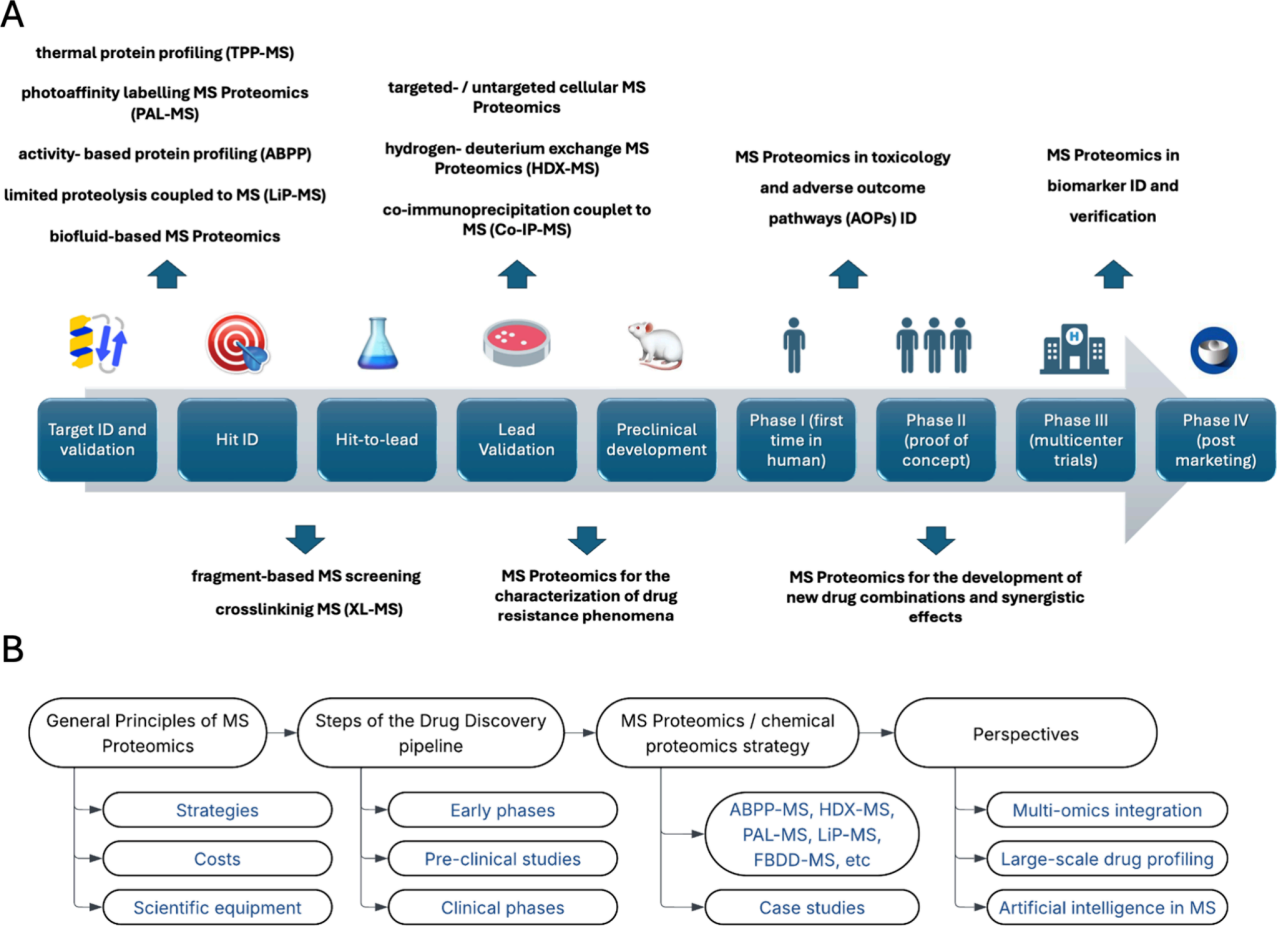
(A) Applications of MS proteomic and chemoproteomic techniques at different stages of the drug discovery process, from the target identification to clinical and post-marketing surveillance. (B) Workflow of the review content.

This perspective provides an integrated overview of the role of bottom-up MS proteomics strategies as integrated in the modern drug discovery pipeline, illustrated in Figure 1, as applied to each stage of the research. We also address recent advancements in MS instrumentation and software tools, which have improved the accuracy and reproducibility of MS proteomics analyses. Finally, we highlight emerging trends and future directions in MS proteomics and chemical proteomics, including their integration with AI and machine learning algorithms, which are expected to further accelerate drug discovery efforts and enhance data interpretation. By bridging the gap between molecular biology and medicinal chemistry, MS proteomics continues to advance our understanding of complex diseases and put the bases for next-generation therapeutics. All these strategies will be discussed in detail with examples throughout the present perspective.

## 2 Principles of MS Proteomics Analysis

Overall, MS proteomics pipelines can be broadly classified into bottom-up and top-down approaches. In bottom-up MS proteomics, proteins are enzymatically digested into peptides, which are then analyzed by MS to reconstruct protein identities and abundances with respect to a control sample.^23^ On the other hand, MS top-down proteomics is used to analyze intact proteins without prior digestion, preserving information about isoforms and modifications but requiring high-resolution instruments due to the complexity of intact protein spectra.^24^ This perspective will discuss only bottom-up MS proteomics applications. Indeed, they are the most often used applications to inform about protein/peptide expression and the most exploited for chemical proteomics purposes these days.

Depending on the purpose of the study, an MS proteomic approach can be defined as either targeted or untargeted. Targeted MS proteomics focuses on the quantitation of specific proteins or peptides of interest.^25^ This approach is often implemented through techniques such as selected reaction monitoring (SRM), multiple reaction monitoring (MRM), and parallel reaction monitoring (PRM).^25,26^ The workflow typically begins with the selection of unique peptide sequences, termed ‘proteotypic peptides’, which represent the target proteins.^26,29^ Following digestion of the protein, these peptides are analyzed through MS. Precursor ions are fragmented, the selected dissociation transitions are monitored according to the peptides of interest, and quantitation is based on peak areas.^27,30^ One of the significant advantages of targeted proteomics is its high sensitivity and reproducibility. Also, by employing stable isotope-labeled internal standards, researchers can achieve absolute quantification of target proteins across various samples.^28^ In contrast, untargeted MS proteomics aims to provide a global overview of the entire proteome without prior knowledge of which proteins may be present or their abundance; therefore, it is an unsupervised approach.^29^ Untargeted proteomics is particularly useful in exploratory or preliminary studies aimed at biomarker discovery, understanding disease mechanisms, or the MoA and off-target activity of investigational drugs.^30,31^ The ability to identify novel biomarkers can lead to significant advancements in personalized medicine by informing treatment decisions based on individual patient profiles.^30,32^ The volume of data generated necessitates robust and multiple statistical methods to ensure that identified proteins are confirmed in the biological samples.^33^ Additionally, issues such as ion suppression and variability in peptide ionization can complicate quantitative analyses.^34^

In the field of untargeted proteomics, two prominent methodologies are “*whole sample”* (e.g., whole plasma, whole cell, and whole tissue) proteomics, also known as “total” proteomics, and fractionated experiments^35^ (Figure 2A). One of the primary advantages of whole sample proteomics is its ability to analyze thousands of proteins simultaneously, making it suitable for large-scale studies.^36^ Whole sample MS proteomics is effective for handling complex mixtures that are difficult to fractionate or separate according to physical or biological characteristics in a reproducible manner without sample loss, such as cell lysates or tissue extracts.^37^ On the other hand, a fractionated MS proteomics experiment includes, at different stages of the preprocessing, a separation of the samples into different subfractions that can be analyzed one by one by the MS to improve instrumental sensitivity and thus protein coverage.^37^

**Figure 2:**
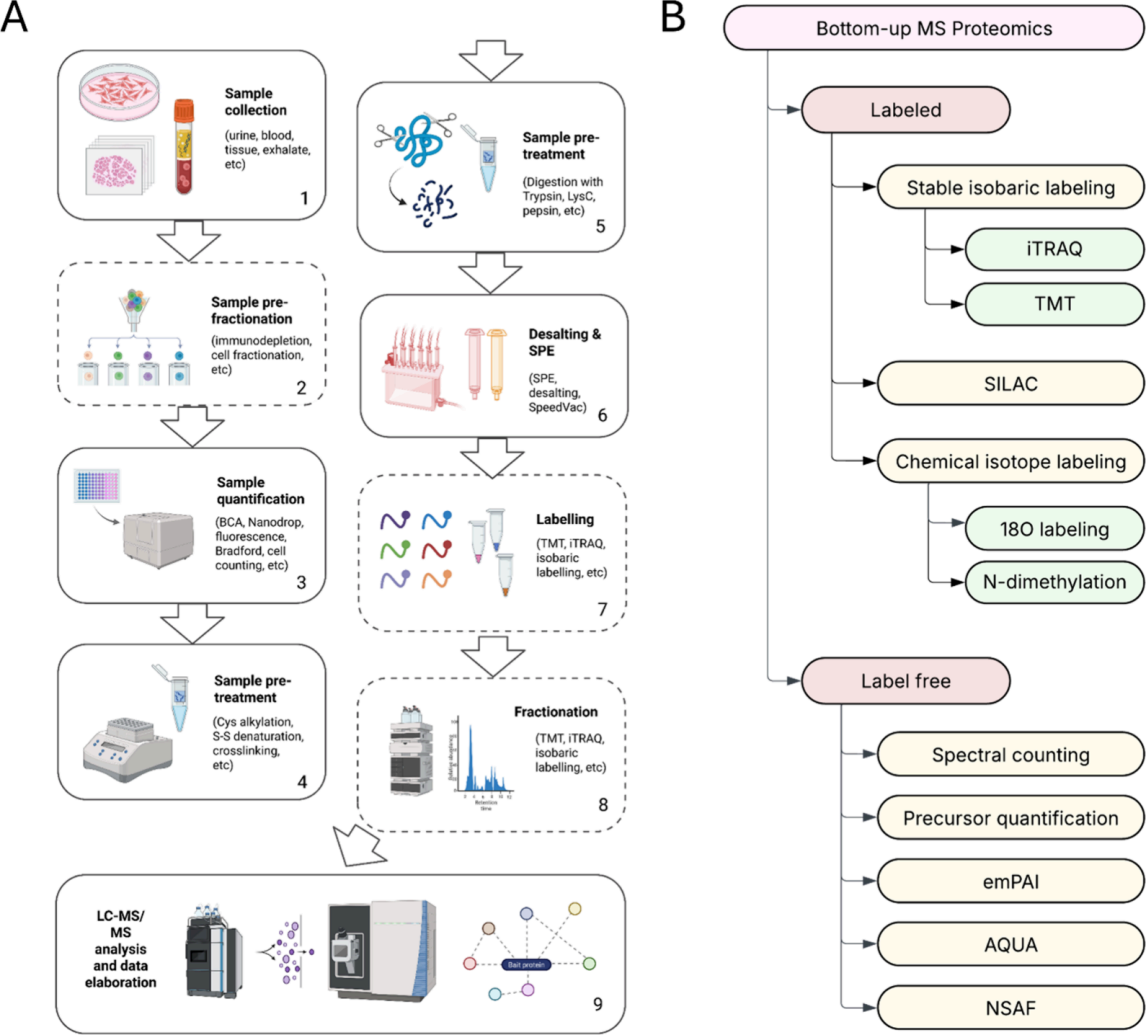
(A) General sample preparation pipeline for a bottom-up proteomics experiment. Steps out of brackets are usually compulsory for any kind of experimental setup, whereas steps in brackets represent optional steps (i.e., for labeling experiments and fractionation experiments). General workflow for bottom-up MS proteomics includes quantification of protein content in each sample specimen to prepare equal volumes of initial proteome, sample preprocessing and digestion into peptides, peptides desalting from organic reactants and salts, and the proper LC–MS/MS analysis. Dashed boxes represent optional steps. (B) General workflows and data acquisition methods for bottom-up MS proteomics experiments. Acronyms are defined as follows: Normalized Spectral Abundance Factor (NSAF), emPAI, AQUA (Absolute QUAntification), TMT Tandem Mass Tag (TMT), isobaric tags for relative and absolute quantitation (iTRAQ), stable isotope-labeled amino acids (SILAC).^38–46^

Examples of fractionation pipelines include serum or plasma immunodepletion to remove the most abundant proteins like albumins or immunoglobulins, which can make up to 80% of the protein content, through the use of immunoaffinity chromatography or cell fractionation of the subcellular compartments (e.g., cytosol from cell nucleus from membrane fraction).^38^ Otherwise, fractionation can occur at the bottom of the digestion protocol, directly on the peptide mixture.^39,40^ In this case, it is usually performed with anionic exchange chromatography (AEX), which allows for peptide separation according to their isoelectric point, resulting in an off-line combined LCxLC for pH and lipophilicity.^41^ Phosphoproteomics is another example of fractionated MS proteomics in which phosphopeptides are purposely isolated and concentrated through ion metal affinity chromatography (IMAC) or TiO_2_ beads, which retain specifically phosphorylated residues and are often combined further with AEX fractionation.^42,43^ Generally speaking, the authors suggest that, unless specific findings are expected from other previous observations, phopshoproteomics is performed only after total proteomics on the same samples. The preparation of samples for bottom-up MS proteomics, either for labeled or label-free pipelines, is a crucial step that ensures the accurate identification and quantification of proteins. Given the diverse objectives of MS proteomic experiments, no universal protocol exists, but a general workflow can be outlined to standardize sample processing, as illustrated in Figure 2A.

### 2.1 Quantification, Peptide Labeling, and Data Acquisition Modes

Quantification approaches are broadly divided into two main categories: label-free quantification (LFQ) and label-based quantification.^44,45^ Both approaches offer distinct advantages and challenges, depending on the experimental goals and sample complexity.^45^ LFQ relies on comparing the intensity of MS signals between different samples to estimate peptide, and thus protein, abundances. This method is straightforward and cost-effective, as it does not require chemical or metabolic labeling of the samples.^46^ There are several types of LFQ pipelines and include spectral counting (SC), peptide ion intensity (PII), normalized spectral abundance factor (NSAF), emPAI, absolute quantification (AQUA).^47^ SC counts the number of MS/MS spectra matched to a particular peptide or protein.^48^ Higher spectral counts are assumed to correlate with a higher protein abundance. Although simple, this method is semiquantitative and may not capture small changes in protein expression.^48^ PII, also referred to as precursor quantification, relies on extracted ion chromatograms (XIC) and measures the intensity of peptide ions (based on their MS^1^ signal) across multiple samples.^45^ The signal intensity of a peptide is proportional to its concentration, allowing for quantitative comparisons, either on the chromatographic peak areas (e.g., PEAKS from Bioinformatics Solutions Inc. or Progenesis QIP from Waters Corp) or to their relative intensity signals (e.g., Proteome Discoverer).^49^ Therefore, this is the most common LFQ method used. The NSAF method normalizes spectral counts by protein length and the total spectral counts in the sample. It reduces bias toward larger proteins and allows more accurate comparison of relative protein abundance across samples.^50^ emPAI, on the other hand, estimates protein abundance based on the ratio of the observed peptides to the theoretically observable peptides for a given protein. This approach accounts for protein size and the complexity of digestion, also providing a semiquantitative estimate of protein concentration.^51^ AQUA method relies on synthetic stable isotope-labeled peptides spiked into the sample as internal standards. The MS signal of the endogenous peptide is compared with that of the labeled AQUA peptide, providing an absolute quantification of protein abundance. While not strictly “label-free” when isotopic standards are used, it is often grouped with LFQ approaches due to its relative quantification principle before normalization.^52^ The absence of expensive reagents such as isotopic labels or specialized equipment makes LFQ a more affordable option for large-scale proteomics experiments, like on large cohorts of samples from patients (>100 patients).^53^ However, the LFQ is more susceptible to experimental variations between runs, such as differences in sample preparation, chromatographic retention times, and ionization efficiency. Such variability can introduce noise into the quantification process.^49^

Labeled proteomics pipeline involves the introduction of isotopic or chemical labels into the sample, which enables the relative or absolute quantification of proteins across different experimental conditions. Labeling can be done either at the protein level or at the peptide level, depending on the labeling strategy.^54^ The three most common labeling strategies are isobaric labeling (e.g., TMT, i.e., Tandem Mass Tag (ThermoFisher) or iTRAQ, i.e., isobaric tags for relative and absolute quantitation (Waters)), stable isotope labeling by amino acids in cell culture (SILAC), and labeling with chemical isotopes.^55,56^ TMT (very similar to the iTRAQ kit) can label up to 32 different samples (32-plex) in a single experiment, allowing for multiplexed quantification. Each tag consists of an MS/MS reporter ion, a mass normalizer, and a reactive group that binds to primary amines on peptides. During MS/MS, the tags release reporter ions of different *m*/*z* values, which are used for relative quantification of the peptides.^57^ The ability to multiplex samples increases throughput and allows for the simultaneous quantification of multiple conditions in a single run. This minimizes run-to-run variability and batch effects.^57^

SILAC, which stands for “stable isotope-labeled amino acids,” is a metabolic labeling technique that introduces stable isotope-labeled amino acids (e.g., ^13^C or ^15^N) directly into the cell culture medium.^56^ Cells incorporate the labeled amino acids into their proteins during growth, creating a direct comparison between “light” (unlabeled) and “heavy” (labeled) peptides in the MS spectra.^58^ It is suitable to study dynamic processes, such as signaling pathways, protein turnover, and interactions in cellular studies, because it allows the incorporation of time points into the experimental design.^56^ On the other hand, SILAC is only applicable to cells that can be cultured in vitro, limiting its use in clinical or environmental samples where metabolic labeling is not feasible.^56^ Another labeling option is the chemical isotope labeling, which involves the use of stable isotope labels that are chemically introduced to peptides after protein digestion.^59^ Common strategies include dimethyl labeling, that introduces dimethylation at the peptide N-termini, lysine residues and generates a mass shift between labeled and unlabeled peptides, or the ^18^O Labeling, which works the same but at the C-terminal carboxyl group.^60,61^ Overall, label-free experiments are preferred in explorative experiments when large groups of samples or large cohorts of patients are being analyzed. This is due to the lower costs associated with sample processing and to the fact that labeled proteomics limits sample size to the one required in vendors’ kit requirements, which is generally a few microliters. On the other hand, labeled techniques in a standard experiment result in higher accuracy and reproducibility, with as low as two replicates per group.^54^ Also, labeling allows multiplexing, resulting in lower costs associated with fewer MS runs needed. Pros and cons of label-free and labeled MS proteomics experiments are reported in Table 1, while a schematic representation of the possible quantification experiments in bottom-up proteomics is reported in Figure 2B.

**Table 1:** Pros and Cons of Label-Free and Labeled MS Proteomics

feature	label-free proteomics	labeled proteomics
sample preparation	simple, i.e., no labeling required	complex as it involves labeling steps
cost estimate	lower for sample preparation (no labeling needed); MS experimental might impact more due to not multiplexing and higher replicate required	higher due to labeling reagents for sample preparation. Multiplexing helps contain costs associated with MS runs
throughput	high, simultaneous analysis possible	moderate (limited to ∼20 samples per experiment)
grouping and replicates	minimum three replicates per group are suggested due to the intrinsic variability associated with label-free workflows	single replicates (as low as 2 samples/group) are enough thanks to high reproducibility and variability
reproducibility	lower, more variability (peptide abundance is quantified on precursor intensity/area)	higher, reduced experimental variability (peptide abundance is quantified on reported ion intensity)
applicability	broad, all biological samples	limited, some techniques restricted to sample type, especially for the minimum amount of material required
flexibility in study design	high, new samples can be added any time before LC-MS/MS analysis	low, fixed once the experimental design has started

Additionally, data-dependent acquisition (DDA) and DIA strategies influence how mass spectra are recorded, balancing the depth of coverage with reproducibility and quantification accuracy. DDA begins with a full-MS scan to detect all ionized peptides in a sample.^62^ From this scan, a limited number of the most intense precursor ions (typically 8–25) are selected in real time for fragmentation, balancing acquisition speed and resolution.^63^ The resulting MS/MS spectra are then used for peptide identification.^62^ While DDA provides high-quality spectra for abundant peptides, it can miss lower-abundance proteins in complex mixtures due to its selective nature. DIA, on the other hand, fragments all precursor ions within predefined *m*/*z* windows, allowing for broader and more consistent proteome coverage.^64^ Instead of isolating individual precursors, DIA divides the full *m*/*z* range (e.g., 400–1200 *m*/*z*) into overlapping windows (e.g., 25 Da each), fragmenting all ions within each window simultaneously.^65^ This results in complex, partially overlapping spectra that require sophisticated computational tools and complete spectral libraries to deconvolute and identify peptides accurately.^66^ A schematic explanation of how DDA and DIA work is illustrated in Figure 3.

**Figure 3:**
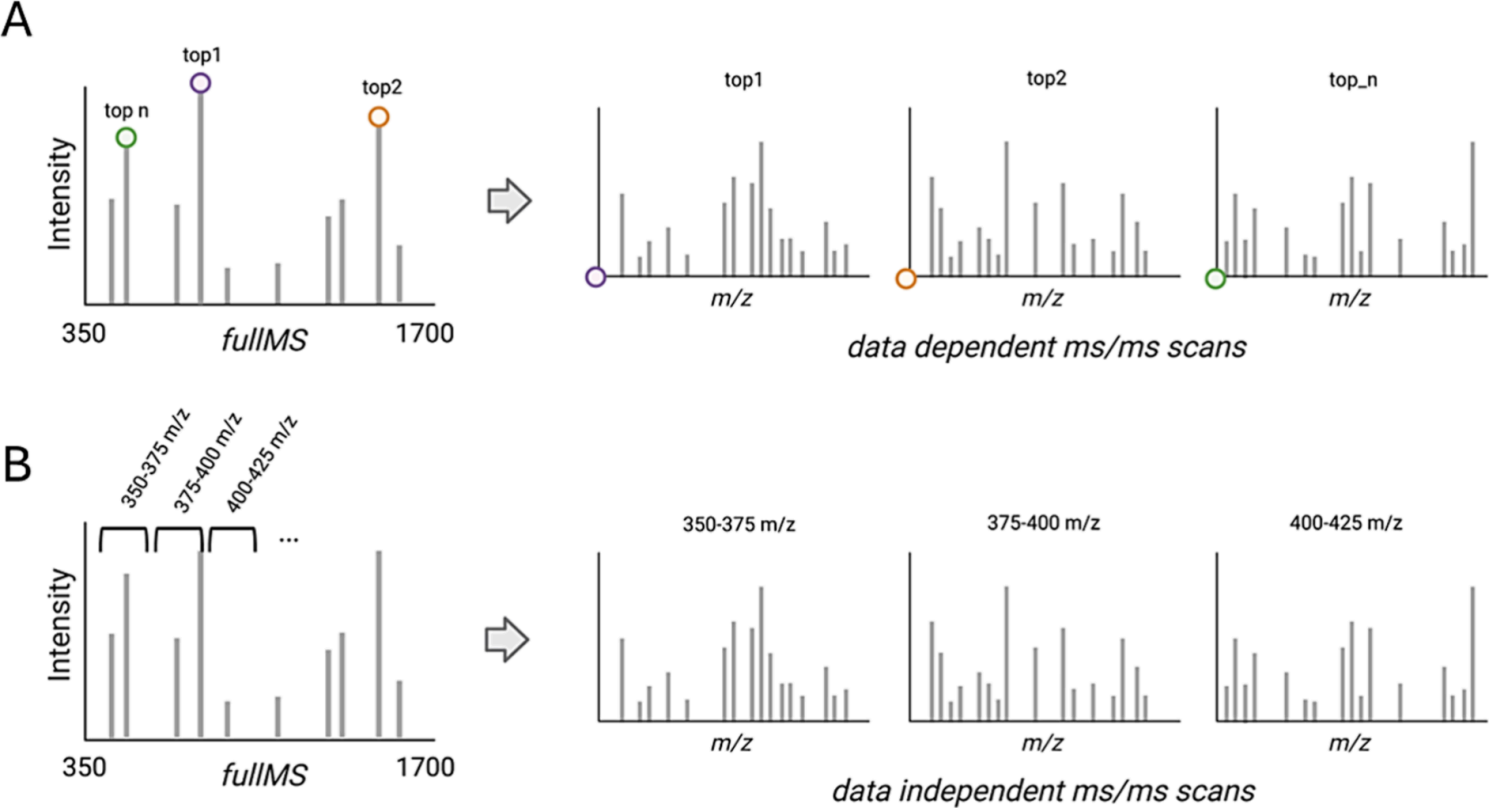
Data-dependent acquisition (DDA) (A) and data-independent acquisition (DIA) (B) schematic workflows. In DDA mode, the top n precursor ions are selected according to their intensities in MS1 scans and fragmented in MS/MS experiments every scan cycle. In DIA mode, the full-MS window is divided into smaller continuous *m*/*z* windows (usually 15–40 *m*/*z*), and all their precursors are fragmented in MS/MS experiments. The overlapping fragment ions for each small window are then deconvoluted by appropriate software.

### 2.2 Current Instrumentations for Bottom-Up MS Proteomics and Operational Costs

All the most modern and cutting-edge models of MS instruments provide high sensibility and, when coupled to nano-LC systems, allow the investigators to perform medium- and high-throughput experimental sessions with minimal operator occupancy and manual intervention required (a comparison between low-, middle-, and high-throughput MS proteomics is provided in Table 2).

**Table 2:** Comparison between Low-, Middle-, and High-Throughput MS Proteomics

feature	low throughput	middle throughput	high throughput
primary objective	deep proteome coverage and discovery of novel proteins/protein isoforms/PTMs	balanced coverage and scalability for comparative studies	rapid and large-scale profiling with high reproducibility
typical use cases	target discovery, pathway mapping, PTM analysis	drug response profiling, target validation, and mechanistic studies	biomarker discovery, patient stratification, clinical screening
sample capacity	<50 samples per batch	50–500 samples per batch	thousands of samples per batch
acquisition mode	usually DDA, sometimes DIA	DIA or multiplexed DDA (TMT/iTRAQ)	DIA or scanning SWATH with shorter gradients
LC setup	nano-LC, long gradients (90 - 240 min)	micro/nano-LC, moderate gradients (30 - 60 min)	high-flow or Evosep LC, short gradients (5 - 15 min)
flow rate	200–300 nL/min (nanoflow)	1–10 μL/min (microflow)	high-flow or Evosep
sample preparation	extensive manual processing; multiple fractionation steps (e.g., SCX, HILIC, high-pH RP)	streamlined digestion and cleanup; optional fractionation	automated digestion and cleanup using liquid-handling robots
throughput (samples/day)	<10	10–50	>100
reproducibility	high within-run precision, lower between runs	moderate to high reproducibility	very high reproducibility (DIA advantage)
limitations	time-consuming, low sample capacity, expensive	intermediate depth, requires optimized LC-MS setup	lower depth, risk of missing low-abundance proteins

High-resolution mass spectrometry (HRMS) analyzers play a key role in MS proteomics, with recent Orbitrap and quadrupole time-of-flight (qTOF) systems being widely used for all the omics disciplines, and recent HRMS dedicated to this purpose.^67^ Orbitrap mass analyzers, from Exploris 480 to the newest Orbitral Astral, which was designed for deep-coverage proteomics workflows, provide increased mass accuracy and resolving power, making them ideal for deep proteome profiling and precise identification of posttranslational modifications.^68^ This is also possible thanks to their ability to distinguish precise isotopic patterns and isotopic ratios, which can be elaborated by proprietary software programs to achieve high selectivity in peptide identification. Also, Orbitrap Astral, introduced in 2023, combines a new electrostatic mass analyzer designed for ultrafast acquisition to ion routing multipole (IRM), to control ion traffic between analyzers. This ensures higher acquisition rates with retained resolution, which results in shorter chromatographic gradients needed compared to “classical” instruments.^68^ qTOF instruments, including the well-known Impact II UHR-QqTOF from Bruker, on the other hand, offer fast acquisition speeds and high sensitivity, especially when combined with ion mobility systems, which are particularly advantageous for discovery proteomics and DIA-based workflows.^69^ The instruments provided with ion mobility (IM) are able to introduce a fourth separation dimension beyond LC, *m*/*z*, and fragmentation, resulting in improved identification of isobaric/isomeric peptides and lower spectral congestion, which is optimal for single-cell proteomics or highly complex samples, for instance. High-resolution MS represents one of the major financial barriers to implementing bottom-up proteomics in drug discovery. The Orbitrap Astral (Thermo Fischer Scientific, Waltham, Massachusetts, USA) costs are typically 1.0–1.2 M$ depending on the regional area, reflecting its cutting-edge speed and proteome coverage. The Orbitrap Exploris 480 (Thermo Fischer Scientific, Waltham, Massachusetts, U.S.), launched earlier, offers a more accessible yet robust solution, with prices generally in the range of $600,000–$800,000 depending on configuration, software packages, and geographical areas (prices are referred to as average for North American and European sellers). Same prices are required to purchase Impact II QTOF (Bruker Corporation, Billerica, Massachusetts, U.S.), Synapt-XS Q-IMS-TOF (Waters Corporation, Milford, Massachusetts, U.S.), and similar instrumentations. Beyond the initial investment, annual operational costs are significant. Indeed, comprehensive service contracts alone often account for 10–15% of the purchase price, while consumables such as LC columns, solvents, labeling reagents, and sample preparation reagents can add thousands of dollars per year in active laboratories.^70^ Staff training and bioinformatics support for large MS data management and storage further contribute to recurrent expenses.^71^ These costs underscore the importance of shared core facilities, consortia, and industry-academia partnerships, which can distribute the financial burden while ensuring access to advanced instrumentation.

## 3 MS Proteomics for Target Identification

The first stage of drug discovery is the target identification and validation stage, which involves biochemical and histopathologic studies on the single proteins or associated pathways involved in the disease onset and progression.^72^ The two main methods to address this stage (i.e., knockout studies or Western blotting) are often slow and limited by the need to focus on a small subset of proteins.^73^ For simplification, these approaches can be divided into genetic-based and biochemical-based. One of the most applied genetics methods in target identification is the identification of a gene causing a resistance phenotype through a phenotypic screening.^74^ Genetic screening can be either direct, aiming to identify the gene underlying phenotype, or reverse, but in both cases.^75^ The biochemical approach to target identification focuses on protein expression and can be addressed through different tools.^76^ The most traditional ones include Western blot, ELISA analysis, and other instrumental techniques, but the emerging role of MS proteomics has started to become central for this stage.^10^ Indeed, MS proteomics allows for comprehensive proteomic profiling, identifying networks that are dysregulated in disease conditions and offering new therapeutic targets.^77^ MS proteomics and chemoproteomics enable a middle- and high-throughput identification of proteins that are differentially expressed or modified in diseased versus healthy states (i.e., prioritize speed, reproducibility, and scalability rather than maximal depth of proteome coverage rather low-throughput techniques).^82^ By analyzing protein abundances and their interaction networks, it is possible to determine potential drug targets that are key to the pathological processes of various diseases.^72^ The two primary proteomics approaches, i.e., shotgun (discovery-based) and targeted proteomics, complement each other in facilitating target identification. Discovery proteomics, also known as untargeted or shotgun proteomics, is especially useful for identifying novel targets in disease contexts where the underlying mechanisms are poorly understood.^78^ This approach allows the identification of thousands of proteins at once, which can then be prioritized for further investigation based on their involvement in disease pathways.^79^

### 3.1 Thermal Proteome Profiling MS

TPP-MS methods combine thermal denaturation with quantitative MS to identify changes in protein stability upon ligand binding or other environmental changes.^80^ TPP-MS relies on the concept that proteins unfold and denature at specific temperatures and precipitate from the sample solution. The processes that can be altered when proteins are bound to small molecules or interact with other proteins or ligands.^80^ By simplifying its description, TPP-MS is a translation of the targeted approach of cellular thermal shift assay (CETSA) onto the whole proteome.^81^ In TPP-MS, a sample, often cell lysate or tissue lysate, is subjected to increasing temperatures to gradually denature proteins.^82^ After heat treatment, the supernatant of the heat-exposed fractions is processed with a standard bottom-up proteomics workflow, and peptide mixtures are analyzed using MS to identify which proteins remain soluble at each temperature, thus being differentially expressed vs the samples not administered with putative ligands.^83^ This is valid under the assumption that the ligand thermodynamically stabilizes protein folding, thus preventing heat-induced unfolding and precipitation.^84^ Besides a specific drug target, TPP-MS can be coupled to bioinformatics tools to identify the biochemical pathways that are involved in the ligand’s MoA through GO’s functional enrichment.^85^ TPP has been employed to identify drug targets in various studies in the last 15 years, thanks to the evolving MS technologies and the emergence of new platforms, either open access or licensed, for MS data organization and analysis.^86,87^ The workflow and principles are illustrated in Figure 4A.

**Figure 4:**
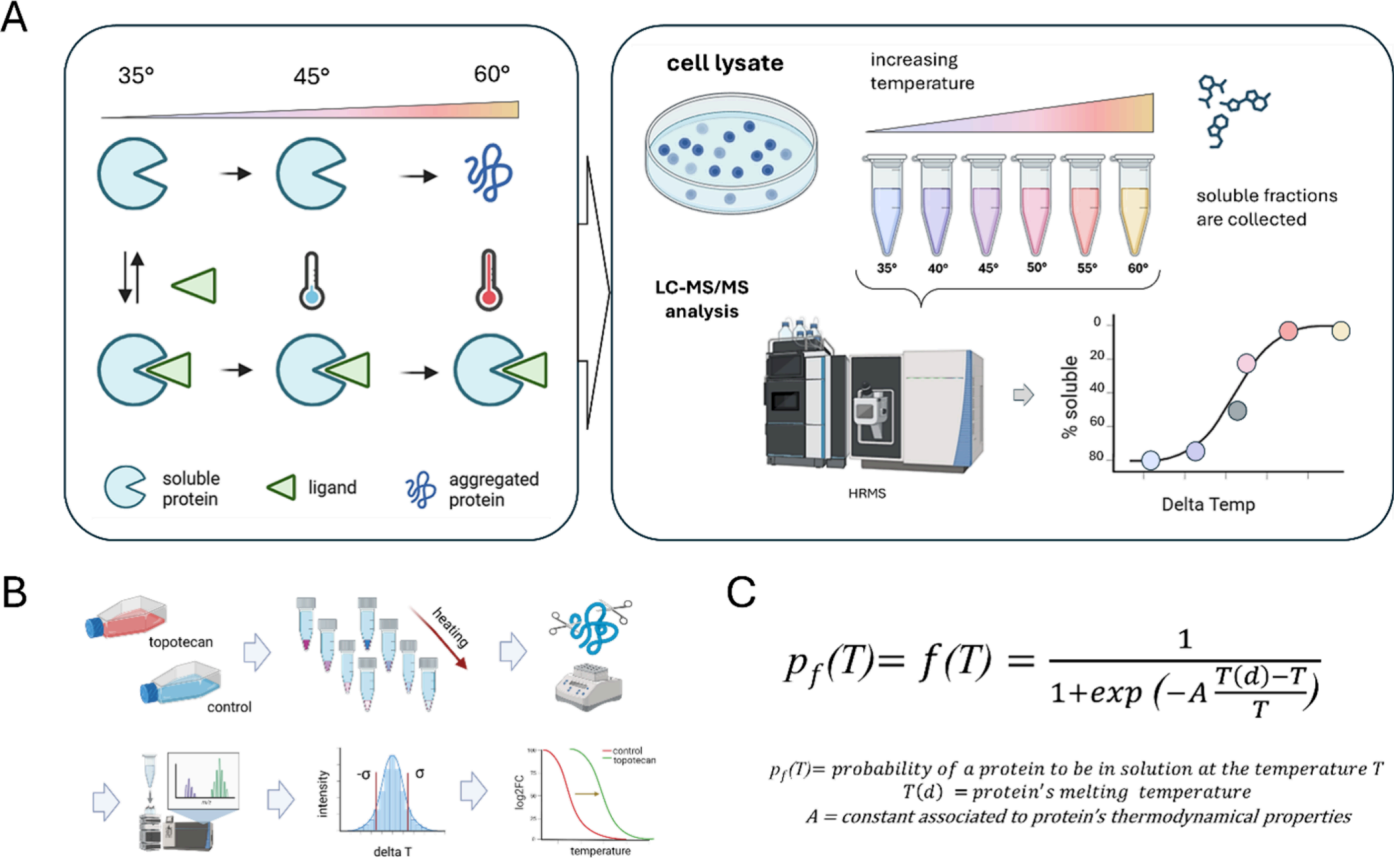
(A) TPP-MS experiment scheme. Adding more compound (ligand) to the protein lysate raises the stability of the target proteins and prevents its aggregation, thus precipitation, even at higher temperatures. The residual concentration of protein–ligand complex in the soluble phase is directly correlated to ligand affinity; thus, *K*_d_ can be extrapolated by a titration curve. The image was generated with BioRender. (B) Example of the application of TTP-MS by Fedorov et al., who exploited TTP-MS to characterize the binding and mechanism of action of topotecan in ovarian cancer cells. (C) Fedorov et al. proposed the equation to allow determining protein solubility curves, derived from the first thermodynamic principles. The target proteins were established to distribute outside 3σ (6.7 °C) of the obtained Gaussian distribution^82^ of the melting temperature reported in the B panel of this picture.

For instance, a recent study from Saei et al. demonstrated the use of TPP to identify thioredoxin reductase 1 (TXNRD1) as a target for auranofin, a gold compound used in treating rheumatoid arthritis.^88^ The study revealed that auranofin binds to TXNRD1, stabilizing it against thermal denaturation, which facilitated the identification of this target through MS analysis and confirmed its on target activity.^88^ By using TPP, Franken et al. demonstrated its utility in identifying cancer drug targets. They used multiplexed quantitative MS to analyze the thermal stability of proteins in K562 cell extracts in response to various kinase inhibitors, revealing direct interactions with specific proteins, including those involved in cancer signaling pathways like the BCR-ABL path, and the AKT protein.^89^ This work highlighted how TPP can elucidate the mechanisms of action for therapeutic agents and identify potential off-target effects.^89^ In another comparative study by George et al., TPP was employed to analyze the stability of proteins in acute myeloid leukemia cells treated with losmapimod, a known inhibitor of the mitogen-activated protein kinase MAPK14 (p38α). The results demonstrated that TPP could effectively detect target engagement of losmapimod with MAPK14 and its downstream target MAPKAPK3.^90^ Similarly, TPP was employed in the Drug Discovery pipeline of nirlotinib for the identification of target/off-target activity by Marín-Rubio et al, that proved that unlike imatinib, it binds directly also to p38α and inhibit the p38α-MK2/3 signaling axis, which suppressed pro-inflammatory cytokine expression and innate immunity markers in activated monocytes derived from acute myeloid leukemia (AML).^91^ In that work, the authors indirectly propose p38α, an off target of the second generation of the tyrosine kinase inhibitors, as new drug target for AML.^91^ TPP was also used for natural products target screening and identification.^82^ This is the example of topotecan, a well-known and characterized TOP1 inhibitor with antiproliferative activity, that was demonstrated to affect the thermal stability of 14 proteins over the established threshold, all of them belonging to DNA replication and cellular metabolism processes.^82^ Workflow performed by Fedorov et al. is illustrated in Figure 4B,C. Overall, TPP-MS offers a more comprehensive alternative to traditional methods, such as differential scanning fluorimetry (DSF) and genetic target validation. Unlike DSF, which requires purified proteins and tests one target at a time, TPP-MS operates directly in complex cell lysates or intact cells, enabling a proteome-wide assessment of ligand-induced stability shifts in a single experiment. This accelerates target deconvolution while preserving the physiological context. Moreover, while genetic knockouts or overexpression systems provide indirect evidence of target involvement, TPP-MS delivers direct, label-free confirmation of compound-protein interactions and selectivity.

### 3.2 ABPP MS Proteomics

ABPP is an MS chemical proteomics technique that has gained significant traction in drug discovery for target validation and MoA elucidation.^92^ ABPP is especially useful for studying enzymes, as it allows profiling their activity in complex biological systems, such as cell lysates or tissue lysates.^93,94^ ABPP involves the use of activity-based probes (ABPs), chemical probes designed to interact with active forms of enzymes, often targeting catalytic or reactive residues with a covalent moiety and a recognition system (i.e., biotin–streptavidin).^92^ ABPPs are highly selective for enzyme families, depending on the reactive group used (e.g., targeting serine hydrolases, cysteine proteases),^95^ and are designed to covalently modify active enzymes, allowing their capture with different recognition systems like biotin–streptavidin and MS analysis.^95^ A general workflow for an ABPP experiment is represented in Figure 5A. ABPP is highly specific for active enzymes (as opposed to merely present proteins), distinguishing between active and inactive or nonfunctional enzyme populations.^96^ By comparing samples treated with a small-molecule inhibitor (or drug candidate) against untreated controls, it can assess how the inhibitor or drug candidate modulates the activity of specific enzymes, enabling target identification and off-target analysis.^96^

**Figure 5:**
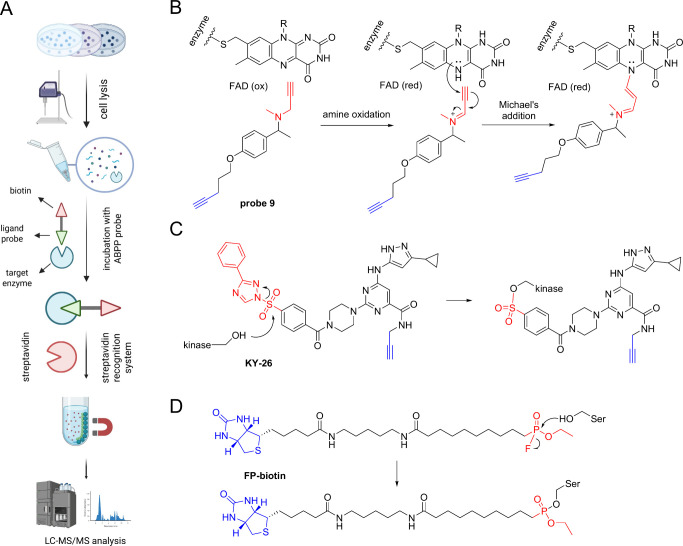
(A) APBB workflow from the cell lysate to MS analysis. The probe is designed so that it targets the catalytic (active) site of the enzyme but cannot be cleaved like the natural endogenous substrate. Thus, the enzyme probe can be fished through a recognizing site, e.g., a biotin moiety, through the biotin–streptavidin system. The image was generated with BioRender. (B–D) Some examples of ABPPs. (A) Probe 9 was synthesized by Krysiak et al. exploited the ABPP strategy to elucidate the enzymatic activity of flavin-dependent oxidases. (B) Probe **KY-26** exploits the electrophilicity of the sulfonamide group to react with tyrosine and lysine residues of several kinases. (C) FB-biotin probe was designed by Tan and colleagues to target clan CA proteases in *P. falciparum*.

Krysiak et al. exploited the ABPP strategy to elucidate the enzymatic activity of flavin-dependent oxidases, in particular monoaminoxidases (MAO A/B).^97^ The probes that the group developed, based on existing MAO inhibitors like selegiline, allowed for selective labeling of enzymes within living cells and offers an effective tool for the drug discovery pipeline of the MAOs both in vitro assays using recombinant proteins and in more complex biological samples such as mouse tissues and human brain cancer cells.^97^ The reaction mechanism of this probe is represented in Figure 5B. In 2017, McCloud et al. successfully exploited the ABPP pipeline to design a pan-kinase probe (**KY-26**, Figure 5C) able to target 133 endogenous kinases even in the presence of high ATP concentrations by targeting a conserved lysine in the ATP-binding site.^98^ Starting from a sulfonyl fluoride-containing probe (XO44 ^99^), the authors synthesized a sulfonyl-triazole analogue that contains a triazole with enhanced leaving group ability in order to modify tyrosine and lysine residues, providing an advanced chemical proteomics method for kinase profiling in live cells, expanding the coverage of kinases beyond previous experiments with noncell-permeable probes like kinobeads. Recently, this target identification approach was employed also to accelerate the drug discovery process of parasitic diseases like malaria and leishmaniases by focusing on cysteine and serine peptidases.^100,101^

Focusing on malaria treatments, Tan et al. introduces new broad-spectrum ABPs designed to target clan CA proteases in *Plasmodium falciparum*, which play a critical role in the malaria parasite’s life cycle and represent an emerging potential drug target.^100^ The authors present new dipeptidic vinyl sulfone probes that can efficiently target both endopeptidases and dipeptidyl aminopeptidases in *P. falciparum* and mammalian cells, with a free N-terminal tryptophan and a fluorophore, which are partially or totally cell permeable^100^ With the aim to explore serine proteases (SPs) in *Leishmania* spp as potential drug targets, given their critical roles in parasite survival and infection, two years later, Porta et al. combined ABPP and (iTRAQ)-based quantitative MS proteomics, as reported in Figure 5D.^101^ A library of fluorophosphonate probes was used to detect and identify active serine hydrolases in different life stages of Leishmania, and two proteins of *L. Mexicana* (carboxypeptidase (LmxM.18.0450) and prolyl oligopeptidase (LmxM.36.6750) were identified as possible druggable targets.^101^ The success and versatility of the ABPP approach to characterize cell proteome, e.g., after the administration of investigational compounds, pushed the bigger chemical suppliers to commercialize standard ABPP kits by encompassing the chemical moiety of the most common protein motifs, including the ATP-binding site of human kinases. For instance, a kit simplification of a kinase-ABPP was employed in the medicinal chemistry studies on gastric cancer (GC) by Choi et al.^102^ In fact, the authors exploited desthiobiotin-ATP, which is able to specifically and covalently target the catalytic lysines of kinase to test the activity of the heat-shock protein 90 (HSP90) inhibitor **AUY922**. Through the whole cell ABPP assay, the microtubule-associated serine/threonine kinase-like (MASTL), known for its involvement in mitotic control and cancer progression, was identified as specifically upregulated by HSP90. Further MS proteomics and bioinformatic studies were employed by the authors to validate this protein as a GC target.^102^ In the context of drug discovery, ABPP-MS is more informative than traditional enzyme assays (e.g., colorimetric or fluorometric substrate-based assays) that measure activity toward one purified enzyme at a time. ABPP-MS can monitor hundreds of enzymes directly in complex proteomes or cells, capturing native context and competition with inhibitors. Also, ABPP-MS is superior and safer to using radiolabeled compounds (e.g., [^3^H]- or [^14^C]-labeled inhibitors) and to “traditional” affinity chromatography/pull-down assays, which rely on immobilized compounds, often yielding nonspecific binders or losing weak/transient interactions, in contrast to ABPP probes that are selective only toward active enzymes.

### 3.3 PAL MS Proteomics (PAL-MS)

Unlike ABPP, which is activity-specific, as it labels only the active forms of enzymes through a covalently stable bond, PAL uses photoactivatable probes, which typically contain a photoreactive group that becomes activated upon exposure to light (usually UV light).^103^ Once activated, this group forms a covalent bond with any protein in proximity.^104^ Unlike ABPP, PAL is not activity-specific. Indeed, it labels proteins based on spatial proximity to the probe rather than their catalytic activity.^11^ PAL probes are generally used to study noncovalent interactions between small molecules (such as ligands, inhibitors, or drugs) and proteins.^105^ PAL probes consist of a photoreactive group (e.g., diazirines, aryl azides, or benzophenones) that is inert until exposed to UV light, a recognition element (such as a drug, ligand, or small molecule) that binds noncovalently to the target protein before photoactivation, and, often, a reporter tag (e.g., biotin or fluorescent group) is also included for detection.^106^ The photoreactive group must be stable under experimental conditions but reactive enough to form a covalent bond with the target upon light activation. Proper placement of the photoreactive group on the probe is crucial.^9^ If the photoreactive group is too far from the interaction site, it may not form a covalent bond with the target, even after light activation. Probes must have balanced solubility to ensure they reach their target, especially if the target is in a hydrophobic environment (e.g., within cell membranes) or a hydrophilic one (e.g., cytosol).^9^ Experimental PAL workflow is represented in Figure 6. PAL probes can capture transient interactions or noncovalent binding events that are often difficult to observe by other techniques.^107^ The specificity in PAL typically comes from the binding affinity of the recognition element (e.g., a drug or ligand) to its target before photoactivation, but once UV light is applied, nearby proteins can also be labeled, resulting in potential off-target labeling.^108^ On the other hand, it requires ad hoc synthetic pathway to obtain a suitable chemical probe encompassing both the UV-activable moiety and the recognition portion (usually, an alkyne able to react with an azide-biotin system through copper-mediated click chemistry).^109^

**Figure 6:**
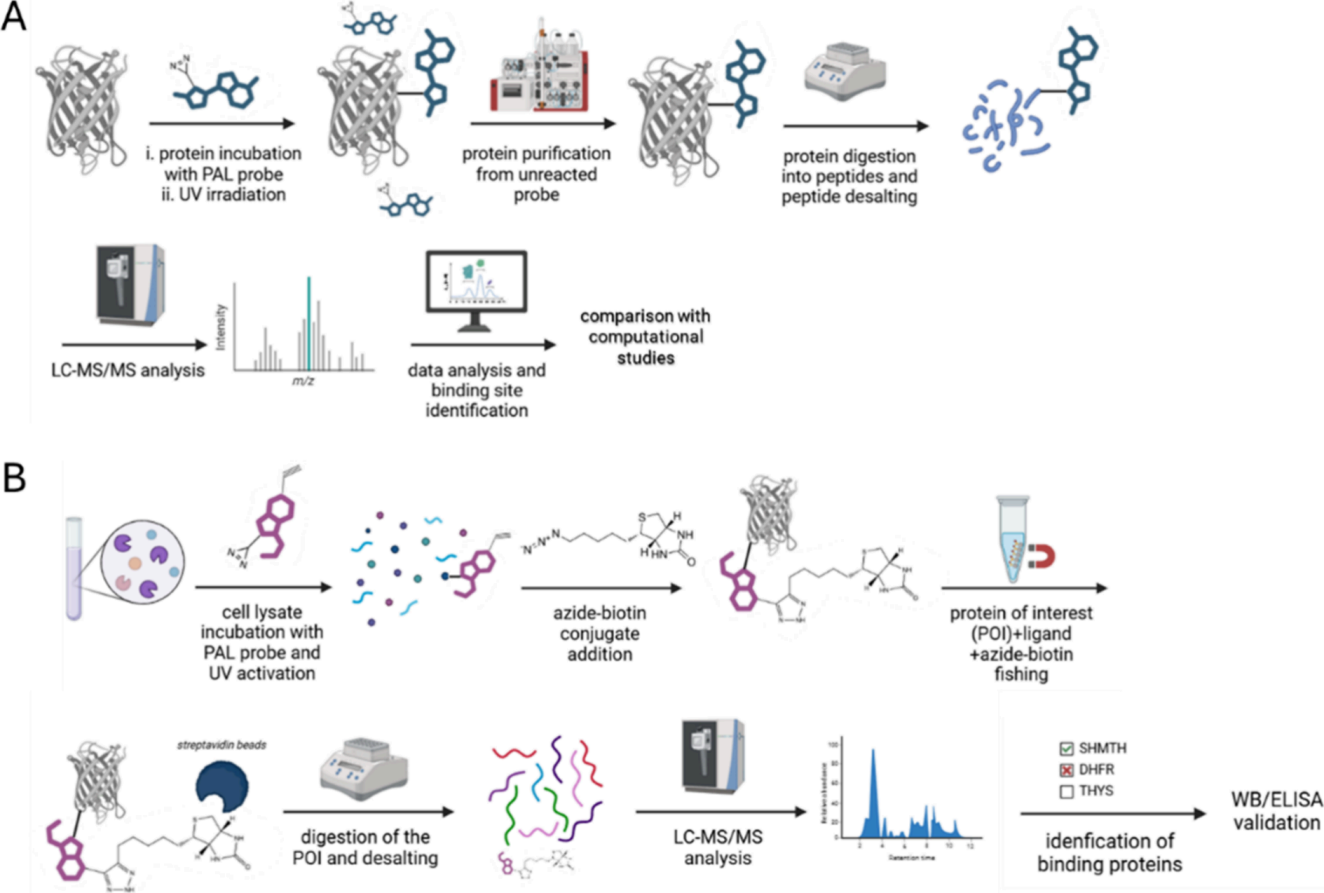
Photoaffinity labeling (PAL) mass spectrometry workflows. A single protein of protein lysate from a cellular experiment (A) is incubated with the PAL probe and photoactivated under a specific UV wavelength and timing. In the case of cell lysate, an azide-biotin bifunctional molecule is added to “fish” the POI and react with the alkyne moiety of the PAL probe. A triazole is formed through click chemistry, and the construct can be recognized with streptavidin beads of affinity columns. (B) Target protein identification in cell lysate. In both pipelines, the experiment includes protein denaturation, cysteine alkylation, hydrolysis into smaller peptides, and MS analysis. The image was created with BioRender.

The chemical design of probes is a critical aspect of PAL, requiring a delicate balance between functional necessity and minimal perturbation of the original drug’s properties. PAL probes must meet specific criteria (as outlined in Table 3) while closely preserving the parent molecule’s chemical structure, affinity, and potency for its target(s) within cellular environments.^110^ This dual requirement applies to both the photoreactive moiety and the pull-down moiety, commonly an alkyne system.^111^ The photoreactive group, whose steric hindrance significantly reduces upon photoactivation, must be carefully selected to avoid altering the drug’s core pharmacophore. Preliminary assays are essential to confirm that the modified probe retains its intended target affinity.^104^ Furthermore, to prevent introducing bias in binding site proximity estimations, the reactive unit should be directly attached to the original drug scaffold, ideally without a linker.^106^ In contrast, the design of the recognition (pull-down) system is more straightforward. Its primary role is to be readily accessible for interaction with the “fishing” system within the cell lysate. Therefore, spacers, such as 2–3 units of polyethylene glycol (PEG) or poly glycines, are often incorporated to enhance its exposure and optimize retrieval efficiency.^104^

**Table 3:** Chemical Requirements of a PAL Probe and the Corresponding Design Considerations to Be Considered during Experimental Planning

component	function	chemical features	design considerations
recognition element	binds selectively to the target protein	derived from the bioactive compound (parent molecule), thus must retain its activity	structure–activity relationship (SAR) must be preserved, i.e. probe should compete with native ligand
photoreactive group	forms covalent bond upon UV activation	small, stable in the dark. Activated by UV (typically 254–365 nm). Generates reactive species (carbene, nitrene, radical, etc)	diazirines and aryl azides are less bulky and more selective. Benzophenone is more stable but bulkier
linker/spacer	minimizes steric hindrance and provides flexibility	chemically inert; variable length; often flexible and hydrophilic	length must allow photoreactive group to reach protein surface without interfering with binding
reporter tag/handle	enables enrichment, visualization and/or detection	must be bio-orthogonal, small enough not to disrupt binding, reactive for click chemistry	tag is often introduced via click chemistry post-labeling to reduce probe size in cell
UV activation wavelength	needed to activate photoreactive group	match the wavelength with the photophore used	choose based on cell/tissue compatibility and minimal biological damage
solubility/permeability	ensures bioavailability and cell penetration	balanced logP. Excessive hydrophobicity or polarity should be avoided	compound should retain the activity of the parent molecule
synthetic accessibility	practical synthesis and modular design	compatible with multistep organic synthesis	avoid labile intermediates or protecting group conflicts
photostability	prevent degradation prior to UV exposure	must remain stable under storage and biological assay conditions	store in dark. Use new stock solutions and photoactivation can occur unintentionally.

One commonly used photoreactive group in PAL is **aryl azide**. Aryl azides are activated by UV light at wavelengths around 250–365 nm, where they undergo a transformation that releases nitrogen gas and produces a highly reactive nitrene intermediate.^112^ Nitrenes can insert into a variety of bonds, such as C–H, N–H, and O–H, allowing the probe to covalently attach to its target. A key advantage of aryl azides is their high reactivity, which makes them suitable for labeling a wide range of biomolecules. However, nitrenes are also short-lived and unstable, which can result in off-target interactions and nonspecific labeling.^113^ Additionally, the need for UV light activation at low wavelengths can damage biological samples, including proteins and nucleic acids, limiting the applicability of aryl azides in certain systems, particularly in live-cell studies, where preserving biological function is crucial.^109^

Another important class of photoreactive moieties used in PAL is **diazirines**. Upon UV light exposure at longer wavelengths (around 350–370 nm) than arylazides, diazirines decompose to produce a reactive carbene species. Carbenes are highly reactive and can insert into C–H and N–H bonds, making diazirines versatile for covalently attaching to a wide variety of targets.^114^ One of the key advantages of diazirines over aryl azides is their longer activation wavelength, which reduces the likelihood of photodamage to biological systems.^111^ Moreover, diazirines are small and compact, meaning they can be incorporated into probes without significantly disrupting the structure and function of the molecules being studied. This makes them particularly useful in labeling complex environments like proteins, lipids, or membranes.^115^ However, alkyl diazirines tend to be less reactive than aryl azides, which means that careful control of light exposure and experimental conditions is necessary to achieve efficient labeling.^116^

A third photoreactive group that is occasionally used in PAL is benzophenone. Unlike aryl azides and diazirines, benzophenones are activated by light at longer wavelengths, typically in the near-UV or visible range (350–380 nm). Upon light activation, benzophenones form a triplet-state excited intermediate that can abstract hydrogen atoms from nearby C–H bonds, generating a covalent bond between the probe and its target. Benzophenones are more selective than both aryl azides and diazirines because they primarily react with C–H bonds, reducing the chances of nonspecific labeling.^117^ Furthermore, their ability to be activated at longer wavelengths makes them ideal for studies in living cells or tissues where minimal photodamage is essential. However, benzophenones require relatively long exposure times to achieve effective labeling, which can limit their utility in fast-acting or dynamic biological processes.^118^ Thus, the choice of photoreactive group must be carefully matched to the specific requirements of the experiment, balancing factors such as reactivity, photowavelength, specificity, and compatibility with the biological system under study. A scheme reporting the three main families of PAL probes and their UV reactivity is reported in Figure 7.

**Figure 7:**
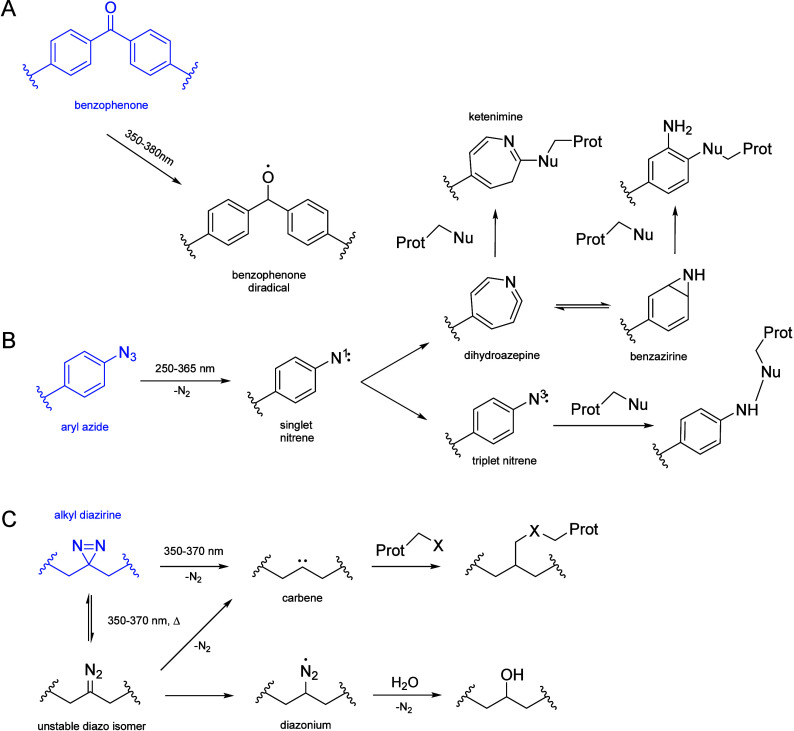
Reaction scheme of the three main classes of PAL photoreactive moieties: (A) benzophenones, (B) aryl azides, and (C) diazirines with their UV activation pathways. On the left, in blue, the initial species is reported.

Since aryl and alkyl diazines are the most used class of photoreactive probes in chemical biology and biochemistry, also outside PAL purposes, their reactivity toward amino acidic residues was recently investigated by West et al., which tested the reactivity of FMOC-protected small aryl and alkyl azides toward equimolar mixtures of N-acetyl, O-methylated natural amino acids.^119^ The authors demonstrate that alkyl and aryl diazirines follow distinct labeling mechanisms. Alkyl diazirines preferentially react with acidic amino acids, such as glutamic acid and aspartic acid, through a reactive diazo intermediate, which is generated prior to carbene formation. This reactivity is strongly influenced by pH, with increased labeling occurring under more acidic conditions, where these residues are protonated and more susceptible to nucleophilic attack. In contrast, aryltrifluorodiazirines primarily label proteins through a short-lived carbene intermediate, which inserts indiscriminately into multiple amino acids but shows a particular preference for cysteine. Unlike alkyl diazirines, the labeling efficiency of aryl diazirines is largely independent of pH, indicating that the carbene pathway is dominant and relatively unaffected by local protonation states.^119^ Proteomic analysis further confirms that alkyl diazirines show a strong preference for labeling membrane proteins, which often possess acidic regions with elevated p*K*_a_ values due to their lipid environment. These findings suggest that the local charge and electrostatic environment of proteins play a crucial role in determining the outcome of PAL experiments and that the choice of the most suitable UV photoactivable moiety determines the good outcome of the experiment.^119^

The PAL technique is more recent than ABPP; thus, its applications are still confined to a limited area of drug discovery research. It has already been employed with success in different fields. For instance, in 2017 Muranaka et al. successfully characterized the exploitable binding sites of the A_2A_ adenosine receptor with PAL, **compound 9**, by exploiting a diazirine-containing chemical probe.^120^ This year, Bon et al. have synthesized a series of oxadiazoline probes that, upon UV activation, generate irreversible diazoketones, which were applied to modify the HDAC1 inhibitor vorinostat (**Probe 7**), the ABL1 inhibitor **Probe 8**, the TMEM16A inhibitor **Probe 5**, and EGFR inhibitor gefitinib (**Probe 9**)^121^ (Figure 10). Their medicinal chemistry was employed to develop **OTX-015** (**MK-8628**) and validate its binding to BRD4-BD1 in a dose-dependent manner without altering binding affinity, resulting in efficient treatment for patients with acute leukemia.^121^ With the purpose of generating a tool to screen the medicinal chemistry of the folate cycle, Takamura et al. successfully designed and tested on *E. coli* lysates a methotrexate (**MTX**) analogue carrying a side spacer linked to the p-aminobenzoic acid of **MTX**, which carry both a UV photoreactive diazirine moiety, and an alkyne for probe fishing (Figure 8).^122^

**Figure 8:**
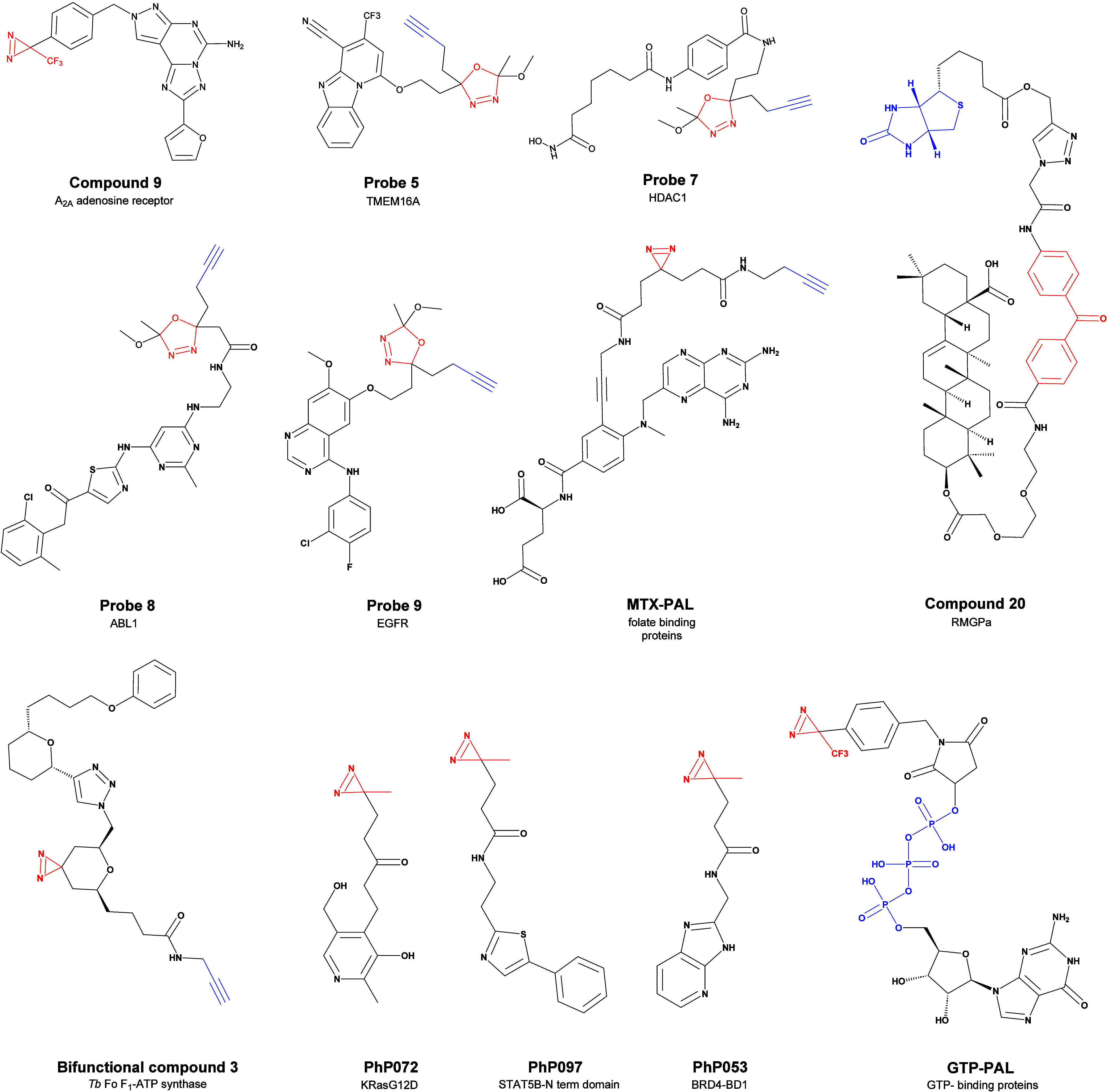
Some examples of PAL probes from the literature with different photoactivable moieties. Blue moieties represent photoactivable fragments, while red moieties are part of the cellular recognition system with the fishing probe, either involved in a covalent mechanism (e.g., terminal alkyne for copper-catalyzed azide–alkyne cycloaddition) or triphosphate for trivalent metals complexation.

The team demonstrated the specificity of the probe versus the folate moiety containing protein, with no binding, even at high concentrations, to albumin and other serum abundant proteins providing a useful chemical biological tool to investigate microbial folate-binding proteins in health and microbiome-related diseases.^122^ Similarly, with a remarkably innovative approach for that time, Kaneda et al. successfully exploited previous ABPP experiments to design of GTP-binding protein (diazirine-based **GTP-PAL**, Figure 9) probes to investigate the selectivity of human GTPases.^123^ In this case, the authors have taken advantage of the triphosphate moiety as a recognition motif for the Fe^III^ IMAC stationary phase. Starting from the same ligand structure, George Cisar et al. exploited the benzophenone derivative with a more common alkyne residue for the same purpose.^124^ Despite the fact that the chemical modification of natural products is more challenging than synthetic-derived molecules, PAL-MS was anyway employed also for the characterization of the pharmaceutical activity of natural products isolated from extracts. This was the case of Zhang et al., who exploited the benzophenone photoactivable moiety to synthesize a derivative of oleanoic acid.^125^ Through this chemical construct, his team investigated all the potential ligands of oleanoic acid as therapeutical targets.^125^ Parallelly, Tulloch et al. developed a natural product library of simplified analogues of acetogenin-type ether and used PAL to investigate the target. Their lead compound, a bis-tetrahydropyran 1,4-triazole inhibitor, was modified into a bifunctional photoaffinity probe.^126^ All of the chemical structures of the examples of PAL here discussed are reported in Figure 8.

**Figure 9:**
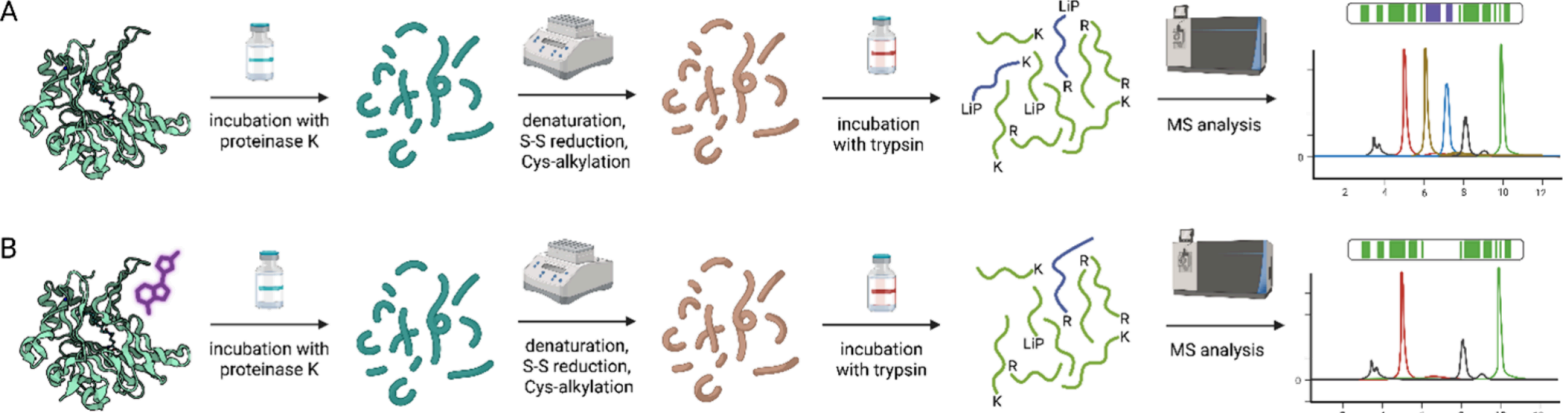
LiP-MS experimental schemes without (A) and with (B) tested inhibitor(s). Cell (or tissue) lysate is then incubated with low concentrations of Proteinase K, thermally denatured, and thiols are reduced with DTT, treated with iodoacetamide, and finally redigested with trypsin to obtain smaller peptides. Resulting peptides are analyzed by LC–MS. Experiments with ligand incubation are compared to control samples, and the least covered sequence regions are identified from a differential analysis.

Through this, the team identified the FoF1-ATP synthase, specifically targeting its α- and β-subunits, as the major target, disrupting the parasite’s ATP production via oxidative phosphorylation.^126^ PAL was also used for FBDD pipelines. Indeed, Ábrányi-Balogh et al. developed a screening approach combining evolutionary-optimized fragment pharmacophores with a diazirine photoaffinity handle, activated by a photocatalyst.^127^ Thanks to this, the authors identified binding sites on BRD4 using a fragment that binds in both the primary acetyl-lysine site and additional sites, discovered new allosteric sites on the oncogenic KRasG12D protein, previously considered challenging to target, and designed a library of fragment hits against the N-terminal domain of STAT5B, which has no known ligands, with evidence of inhibiting cancer cell viability.^127^ PAL and ABPP are central tools in chemical biology. While PAL is suitable for mapping noncovalent binding interactions between small molecules and proteins through proximity-based photolabeling, ABPP is uniquely suited to probe functional enzymatic activity by covalently modifying active-site residues of enzymes in an activity-dependent manner. Their distinct but complementary mechanisms make them powerful strategies for elucidating target engagement, mechanisms of action, and off-target profiles in complex biological systems. Their comparison is reported in Table 4. Overall, PAL-MS represent a solid and more valuable alternative to single amino acid mutation (like alanine scanning) and mutant mapping for target ID and binding site description. Compared to these older techniques, PAL-MS results are more robust, i.e., also allowing to work with the intact protein, and therefore the system is more adherent to the biological system represented. Also, considering time needed to purify and test a library of a protein mutant, despite not being a short-lasting experiment, PAL-MS may faster these preliminary processes. Furthermore, when combined to MD and computational models, as in the previously mentioned works, it represents a valuable tool for the early phases of drug discovery.

**Table 4:** PAL’s Strength and Limitations in Comparison with the ABPP Technique

feature	ABPP	photoaffinity labeling (PAL)
mechanism	activity-based covalent labeling of enzymes	proximity-based covalent labeling via UV activation
specificity	highly specific for active enzymes	depends on the binding affinity of the recognition element, i.e. less specific after UV activation
aims	enzyme profiling, target identification, mechanism of action studies	mapping noncovalent drug–protein interactions, studying protein complexes
proteins studied	enzymes	no limitations
labeling type	covalent modification of active sites	proximity-based cross-linking after UV activation
advantages	functional insight into enzyme activity	captures transient interactions and studies nonenzymatic proteins
limitations	limited to enzyme profiling	potential nonspecific labeling at high μM *K*_d_’s. Chemical probe might be nonsynthetically accessible

### 3.4 Limited Proteolysis MS

LiP coupled to MS (LiP-MS) has recently emerged as a highly informative chemoproteomics strategy to study target identification and binding site characterization.^128^ Its principle lies in the fact that ligand binding alters the structural dynamics and accessibility of proteins, as in their folded state they are only partially accessible to proteases, and exposure of flexible regions or pockets determines cleavage susceptibility.^129^ When a small molecule interacts with a protein, the induced conformational stabilization or rearrangement changes the landscape of protease accessibility, thus modifying the proteolytic cleavage pattern. LiP-MS leverages this principle by applying a controlled, partial digestion of the proteome, followed by global peptide analysis with MS, to detect changes in proteolytic fingerprints between ligand-treated and untreated samples.^130^ These differential patterns are then interpreted as signatures of direct or indirect molecular engagement. An LiP-MS experiment begins with the preparation of a biological system, very often cell culture lysate or an organelle, in which protein conformation is maintained in a near-native state.^131^ The proteome is divided into two or more parallel samples, one exposed to the compound of interest (often at increasing concentrations) and the other serving as a reference control.^132^ The key step is the application of a nonspecific protease, most commonly proteinase K, under mild/diluted conditions and controlled temperature, which ensures LiP rather than complete digestion.^133^ The enzyme is allowed to act briefly, long enough to generate cleavage at solvent-exposed and flexible regions but not to degrade proteins entirely. This step generates structurally informative cleavage products, the distribution of which depends sensitively on the conformational state of the proteins.^134^ The reaction is then quenched with a reducing agent, remaining proteins/peptides are denatured, and the resulting protein fragments undergo a second digestion with a site-specific protease such as trypsin, which standardizes peptides for MS detection and quantification. Quantitative analysis then identifies peptides whose abundance significantly differs between the two conditions, namely, conformotypic peptides.^135^ Unlike affinity-based methods, LiP-MS does not require derivatization or immobilization of the ligand, which can alter its binding properties or introduce artifacts. It also circumvents the need for engineered protein tags, which are not always feasible in complex systems or clinical samples. All these premises make LiP-MS cheaper in terms of economic resources and time spent on sample processing. Because LiP-MS reports on conformational changes, it goes beyond mere detection of binding and can capture allosteric effects or secondary structural rearrangements that are invisible to classical pull-down approaches.^136^ Nevertheless, several limitations must be recognized. Proteins expressed at low abundance or proteins with few solvent-exposed protease cleavage sites may not yield a sufficient signal to detect changes. The method also requires careful optimization of protease concentration and digestion time.^133^ Overdigestion can obscure site-specific effects by generating excessive fragmentation, whereas underdigestion reduces the discriminatory power of the assay. Moreover, not all observed proteolytic differences directly correspond to ligand binding. Some may reflect indirect consequences such as protein stabilization within a complex, conformational shifts caused by downstream signaling, or even protease preference biases.^133^ A schematic representation of an LiP-MS general experiment is depicted in Figure 9.

LiP-MS was successfully used in numerous medicinal chemistry programs aimed at the identification of drug targets. In particular, but not limited to, after phenotypic screening and with vegetal extracts with pharmaceutical properties. This is the case of Zhao et al., who found that cinobufagin can revert bortezombib resistance in myeloma cells by promoting SEC62 expression, thus its association with the partner TRPM4, that resulted in the activation of necrosis by sodium overload (NECSO) pathway. In particular, the authors demonstrated Cinobufagin dose-dependent binding to TRPM4 by combining MD with LiP-MS performed on 8226-BTZR cells.^137^ Similarly, Yong-Chun et al. demonstrated through LiP-MS that Sanguinarine halts oral squamous cell carcinoma by binding to PKM2/TFEB. The researchers treated CAL27 cells with three different concentrations of sanguinarine, pretreated with Proteinase K, and with pancreatic lysate enzymes after denaturation. The only six proteins that resulted as differentially expressed between treated cells and control cells were considered putative targets. Among them, PKM2 had the lower p-value, indicating that it is the preferred target.^138^ In the field of marine drugs, Gracilioether A was found to be a micromolar inhibitor of USP5 (ubiquitin carboxyl-terminal hydrolase 5) by DART (drug affinity-responsive target stability) combined with LiP-MS. The authors exploited the former technique to perform target identification studies, while LiP-MS was used here to decipher the Gracilioether A binding site to USP5.^139^ With the same workflow, the same group also identified the polybrominated Mycalin A as a GRP75 inhibitor, with consequences on p53 cytosolic retention and cell survival.^140^

Overall, TPP-MS, PAL-MS, ABPP-MS, and LiP-MS represent four valid chemical proteomics strategies for target identification and other steps of the drug discovery pipeline. They all possess pros and cons according to the aim of the research context, and their use must be carefully evaluated also according to the financial resources dedicated to the considered project, the available scientific expertise (e.g., despite its precious outcome, PAL-MS strictly necessitates a solid organic chemistry expertise and equipment, and TPP-MS requires physical chemistry expertise to obtain insight into thermodynamical data), and the available time. Furthermore, all these MS chemoproteomics techniques require a preliminary phase to optimize the working conditions. A brief comparison of the prerequisites, time, and costs associated with each technique is illustrated in Table 5, intended as a simplified preliminary guide to choose the best experiment.

**Table 5:** Comparison of Prerequisites, Time, and Costs Associated with TPP-MS, PAL-MS, ABPP-MS, and LiP-MS

technique	economic cost	scientific expertise needed	time needed	software commonly used
TPP-MS	medium-high (many MS experiments, reagents, cell cultures, etc.)	medium-high (thermal shift expertise required and physical-chemical interpretation of results)	medium-long (several days experiment + data analysis), ∼1–3 weeks	TPP-Analyzer (R), MaxQuant, Perseus, FragPipe
PAL-MS	high (probe synthesis + probe tests, MS experiments)	high (chemical probe design + proteomics)	long (weeks for probe design, test, and synthesis, days for experiment), >1 month	MaxQuant, Proteome Discoverer, Skyline, in-house scripts
ABPP-MS	high (activity probes + enrichment + MS experiments)	high (chemistry for probe design + MS proteomics)	medium-long (probe synthesis + days for experiment), ∼1 month	MaxQuant, Proteome Discoverer, custom R/Python scripts
LiP-MS	medium-low (protease + MS experiments, no probe synthesis needed)	medium (proteomics expertise, protease optimization)	medium-short (few days), ∼1–2 weeks	MSFragger, FragPipe, MaxQuant, Skyline

### 3.5 Biofluid-Based MS Proteomics

Biofluids, such as plasma, serum, cerebrospinal fluid (CSF), urine, and saliva, provide a preclinically accessible and usually little invasive source of information for target identification in Drug Discovery.^141^ In contrast to cell or tissue proteomics, which often requires invasive procedures and is limited to a single time point, biofluid MS proteomics allows longitudinal sampling and can represent a systemic view of disease-associated protein dysregulation that can be used for novel drug target investigations.^142^ These features make biofluid proteomics particularly suited for translational applications, where the discovery of novel therapeutic targets must be aligned with clinical feasibility.^143^ The principal challenge of biofluid-based MS proteomics is related to its intrinsic dynamic range of protein concentrations.^143^ Plasma, for instance, contains proteins spanning over 10 orders of magnitude in abundance, with albumin and immunoglobulins accounting up to 80% of total protein content.^144^ Such highly abundant proteins (namely, HAPs) frequently mask low-abundance proteins (LAPs) that may carry greater biological relevance as disease-associated targets.^144^ To overcome this limitation, workflows typically include immunodepletion of the most abundant proteins, often through affinity chromatography columns, followed by fractionation strategies to reduce sample complexity.^145^ These solutions are usually built to remove either only albumin and immunoglobulins or to include antibodies also toward the complement and coagulation system.^146^ Applications of fluid-based MS proteomics for target identification are broad. Under oncology and chronic inflammatory conditions, plasma proteome profiling has uncovered dysregulated proteins of complement and coagulation cascades, which serve as potential therapeutic targets as well as biomarkers for disease progression. This is the example of Mazidi et al., who used plasma-based proteomics to identify drug targets for ischemic heart disease (IHD).^147^ The authors initially identified 13 proteins that showed potentially causal associations with IHD, including N-terminal prohormone of brain natriuretic peptide and proprotein convertase subtilisin/kexin type 9. The combination of MS proteomics results with transcriptomics showed that FURIN is a potential novel target and matrix metalloproteinase-3 a potential repurposing target for IHD.^147^ CSF MS proteomics also has become an indispensable tool in neurodegenerative research, where altered levels of synaptic, axonal, and myelin-associated proteins provide both mechanistic insight and candidate drug targets for diseases such as Alzheimer’s disease, multiple sclerosis, and other neurodegenerative conditions.^148,149^ This is the main purpose, for example, of the Global Neurodegeneration Proteomics Consortium (GNPC, https://www.neuroproteome.org/), a public–private partnership to build one of the world’s largest harmonized proteomic data sets, which now contains protein measurements from multiple platforms from more than 35,000 biofluid samples from CSF and plasma.^150^ Urine proteomics has been particularly valuable in nephrology drug discovery, where proteins associated with tubular dysfunction or glomerular injury not only serve as indicators of drug-induced nephrotoxicity but also reveal novel pathways involved in disease progression, as in the case of chronic kidney disease (CKD).^151^ More recently, salivary proteomics, either whole cell or cell-free, has gained traction as a noninvasive method to identify systemic immune alterations, demonstrating that even peripheral fluids can be informative for target discovery.^152^

## 4 MS Proteomics for Hit Identification and Validation

### 4.1 MS-Guided FBDD

Hit identification is a critical step in the early stages of drug discovery, where molecules with biological activity against a target are identified from large chemical libraries.^153^ MS-based proteomics facilitates hit identification by providing insights into the molecular mechanisms of potential drug candidates.^153^ For instance, MS Proteomics has become a key tool in the FBDD process, through high specificity in detecting fragment-target interactions, elucidating the binding mechanisms, and validating hits at an atomic level besides X-ray crystallography.^154^ FBDD is a powerful approach used to identify small chemical fragments that bind to a biological target.^155^ These fragments are typically much smaller than conventional drug-like molecules, containing fewer than 20–30 atoms.^156^ Once these fragments are identified, they are optimized into more potent compounds through various strategies.^157^ FBDD offers several advantages over X-ray crystallography or other techniques, including higher hit rates (smaller fragments are more likely to bind due to their reduced complexity), better chemical space exploration, as fragments represent a broader range of chemical diversity, and efficient optimization, as MS identified fragments serve as starting points to grow into potent, drug-like molecules.^158^ FBDD has successfully been used in the discovery of several approved drugs (e.g., vemurafenib for cancer and venetoclax for chronic lymphocytic leukemia).^159,160^ Native MS is a powerful technique for this purpose because it allows for the direct detection of intact protein–ligand complexes without requiring labels or complex secondary reagents.^161^ In native MS, the protein and fragment are ionized under conditions that preserve the noncovalent interactions.^79^ When a fragment binds to a protein, the mass of the resulting protein-fragment complex increases, which can be directly measured by a mass spectrometer. The comparison between the mass of the unbound (apo) protein and the bound (holo) protein-fragment complex provides evidence of binding.^162^ Additionally, native MS can detect multiple fragment-binding events if the protein has more than one binding site, offering insights into the stoichiometry of fragment binding.^163,164^ For example, if a protein has two identical binding sites, native MS can show whether one or both sites are occupied by fragments, which is crucial information for subsequent fragment optimization and drug design.^12^ To facilitate high-throughput fragment screening, pooling strategies are sometimes used in MS-based FBDD. Small libraries of fragments are pooled together and incubated with the target protein, and MS is used to detect which fragments bind.^79^ In this strategy, MS can deconvolute the pooled fragments to determine which specific molecules are responsible for binding.^165^ An example of FBDD through native MS is the exploration of CYP121 as a target for drug development against antibiotic-resistant strains of *Mycobacterium tuberculosis* by Kavanagh et al., who combined X-ray crystallography, native MS, and ITC to discover a new hit compound, **25a**, with an affinity of 15 nM for CYP121.^166^ The authors began their research using X-ray screening of low molecular weight fragments. These fragments are small molecules, and their initial binding to the recombinant protein was relatively weak in the mM affinity range. Through a process of merging (linking) these fragments, they synthesized a new series of 5-amidopyrazole derivatives. To assess the improved binding affinity of these new compounds, they tested them in a dose-dependent manner using native MS. This method allowed them to precisely determine the 5-amidopyrazoles binding affinity by analyzing the dose dependency of the concentration of MS complex generated ultimately helping them identify the most effective antibacterial agent.^166^ With a similar approach, Vaaltyn et al. identified a small cohort of molecular fragments whose binding to the TPR2AB region of the target, HOP inhibits the complex formation between HOP and the HSP90 C-terminus.^162^ Their experimental observations lead to the discovery of losartan as a weak but consistent HSP90 inhibitor.^162^

### 4.2 Cross-Linking MS Proteomics

Cross-linking (XL)-MS is an MS technique used to investigate protein–ligand interactions, including fragment binding in FBDD. XL-MS involves the use of chemical cross-linkers that covalently bind specific amino acid residues that are spatially close in a protein or between proteins.^167^ When applied to FBDD, XL-MS can provide valuable structural information about how a fragment interacts with its protein target, including identification of the binding site, conformational changes upon fragment binding, and insights into the overall architecture of the protein or protein complex.^168^ XL-MS involves the use of chemical cross-linkers that react with specific amino acid side chains, such as lysine, cysteine, or serine, to covalently link them if they are within a certain distance from one another (typically 10–30 Å).^169^ When a fragment binds to a protein, it can induce conformational changes, stabilize certain regions of the protein, or bring specific amino acid residues into proximity. The resulting cross-linked products are digested into peptides, analyzed by MS proteomics, and mapped onto the known or predicted protein structure.^170^ XL-MS works by utilizing cross-linkers that are bifunctional chemical reagents capable of covalently binding two amino acid residues within a defined distance range. The cross-linking process is driven by the proximity of the reactive residues when a protein assumes a particular conformation, making it an effective tool for capturing dynamic interactions.^171^ Cross-linkers come in various forms, including the homobifunctional cross-linkers, i.e., reagents that contain two reactive groups that target the same type of residue (e.g., two lysines), heterobifunctional cross-linkers, which have two different reactive groups that can target different types of residues (e.g., lysine and cysteine), and cleavable cross-linkers, which are designed to be cleaved under specific conditions (e.g., reduction, UV light), making it easier to identify the cross-linked peptides in the MS.^172^ An example of XL-MS in FBDD is represented by the hit identification process of innovative thymidylate synthase (TS) destabilizers with anticancer activity described by Costantino et al.^173^ In their drug discovery pipeline aimed at finding innovative allosteric thymidylate synthase (TS) inhibitors, the authors used MALDI-TOF and LC-ESI-MS to investigate the affinity of a TS mutant enriched with cysteines (TS C195S–Y202C variant) vs a library of thiols and disulfides. More than 80 reactive compounds were tested, and this strategy allowed to identify two bioisosterically analogues of the bound fragments, as hit compounds, namely **B12** and **B26**, were optimized and used to develop the lead compound **E7**.^173^ The workflow is illustrated in Figure 10.

**Figure 10:**
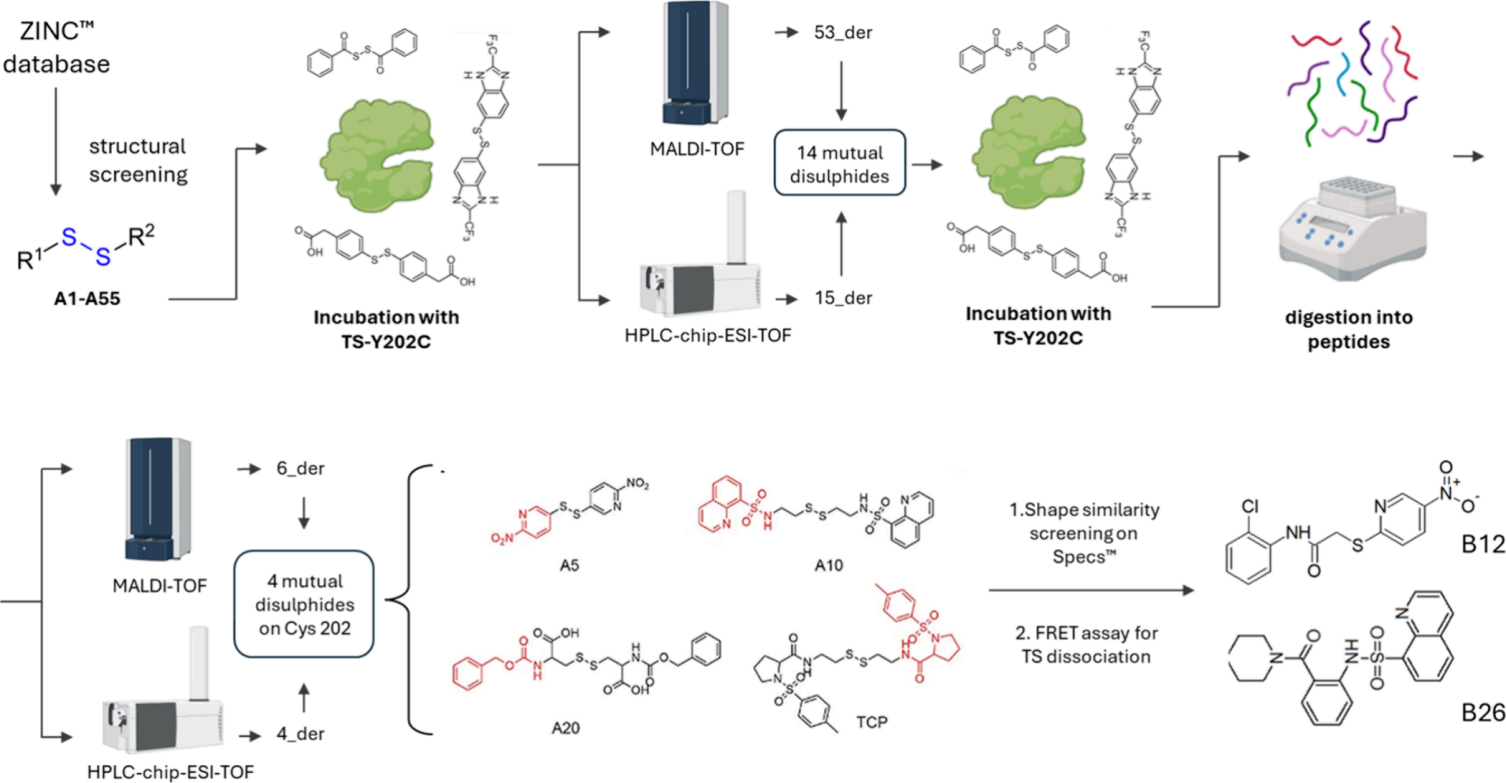
Experimental pipeline used by Costantino and colleagues^173^ to map reactive cysteines on mutated TS surface. The authors exposed the TS C195S-Y202C variant to a library of 55 disulfides from a larger database in ZINC and analyzed both derivatized intact protein with MALDI-TOF in a top-down experiment and in HPLC-chip-ESI-TOF. The candidates that were found bound in both MALDI-TOF and ESI-TOF experiments (*n* = 14) were digested into peptides with ESI-MS and submitted to sequencing analysis to evaluate Cys180, Cys199, Cys202, and Cys210 binding. Only disulfides that bound selectively to Cys202, not involved in catalytic pocket reaction nor in mRNA interaction, were selected for optimization (*n* = 4). Those fragments were submitted to shape similarity on the Specs data set. The best 25 candidates (**B1**–**B26**) were submitted to the FRET experiment for their ability to dissociate TS, and **B12** and **B25** were selected as the best hit compounds. This allowed to develop compound **E7**, a TS destabilizer lead compound.^173^ The image was created with BioRender.

With a similar approach, Fan et al. investigated the acylation pocket of the transcriptional enhancer-associated domain 2 (TEAD2) with a library of small ligands similar to the previously identified flufenamic acid, containing an acrylamide warhead able to be attached by Cys380.^174^ MS proteomics displacement assays lead to the identification of the hit compound **MYF-01–037** as highly affine to the acylation pocket, which led to the identification of the lead compound **MYF-03–069**, active in cells in the nanomolar range with an optimal PK for oral administration.^174^ Despite still being a bit underused compared to other techniques, the integration of FBDD and cross-linking proteomics with MS provides a technically advanced platform for mapping ligand–protein interactions with high sensitivity and resolution. In contrast to traditional FBDD methods, such as NMR, X-ray crystallography, or SPR/GCI, which require purified proteins and large sample quantities and often overlook low-affinity or transient binders, MS-based proteomics enables fragment screening directly in complex lysates or intact cells. This allows the identification of interactions in the native proteomic context. XL-MS enhances this further by covalently stabilizing weak fragment-protein complexes through reactive linkers, facilitating subsequent MS-based localization of binding sites at the peptide or residue level. These combined strategies provide a quantitative, proteome-wide view of ligand engagement, extend chemical coverage to traditionally “undruggable” targets, and accelerate hit validation and MoA studies compared with conventional structure-based or biophysical screening techniques.

## 5 MS Proteomics for Lead Optimization: MoA and Off-Target Activity

MS proteomics contributes to the refinement of drug candidates by helping define the SAR, which correlates changes in the chemical structure of compounds with their biological activity.^21^ MS proteomics allows researchers to rapidly assess how modifications to a compound affect its ability to bind to the target protein, its efficacy, and its specificity.^21^ This accelerates the identification of lead compounds with the best activity and lowest toxicity.^21^ Once validated, hits undergo lead optimization, where the chemical properties of these compounds are refined to enhance their PK and PD characteristics.^175^ MS proteomics continues to play a vital role in this process and is often exploited during these phases as label-free MS proteomics or TMT/iTRAQ labeled to gain insight into the general mechanisms of action identification and as hydrogen–deuterium exchange MS (HDX-MS) to characterize the conformation of a target protein that changes upon binding a ligand.^176^ Also, coimmunoprecipitation followed by MS (Co-IP-MS) is another technique that plays a central role in hit and lead development up to the preclinical stages. Indeed, it allows us to quantify the impact of a drug candidate on protein complex formation; thus, it represents a gold standard in the development of PPI inhibitors.^177^ During the stages of drug discovery, time-resolved experiments (i.e., comparing different time points after compound administration) or dose-resolved experiments (i.e., comparing different compound doses) are often used to elucidate different biochemical pathways involved in their mechanisms of action.^178^

Recently, Lee et al. published an article on time-dependent changes in the proteomes of cancer cells as they expand. These changes, particularly in cell division, adhesion, and metabolism, begin as early as the second day of culture.^178^ The study uses label-free MS proteomics to screen a library of natural products and identifies four pentacyclic triterpenes that are effective against cancer cells under prolonged growth, and it represents a good example of time-resolved exploitation of MS proteomics for broad-range MoA investigation.^179^ The authors discovered that some tested compounds selectively target highly overgrown cells, offering potential avenues for treating drug-resistant cancers, and they demonstrated that caution should be taken in the evaluation of anticancer drugs using differently old tumor cells, introducing the concept that some compounds are stage-specific.^179^ Last year, Chang et al. explored how lysine deacetylase inhibitors (KDAC inhibitors or KDACis) affect cellular processes at the proteome, acetylome, and phosphoproteome levels through dose-dependent proteomics.^180^ The research systematically profiles the impact of 21 KDAC inhibitors (KDACis), revealing that many acetylation sites respond to these drugs in a dose-dependent manner, and concluded that KDACis, especially HDAC1/2/3 and HDAC6 inhibitors, share many common acetylation targets. Through this study, the authors offer a valuable resource for investigating drug mechanisms in different subtypes of cancers and propose a new MoA for panobinostat, which also induces cytosolic accumulation of the paralogous lysine acetyltransferase KATs p300 and of the CREB-binding protein CBP.^180^ With a similar purpose, through the use of Functional Identification of Target by Expression Proteomics (FITExP), which consists of a differential MS-based proteomics analysis of cells treated with inhibitor(s) vs control cells, Saei and colleagues deciphered the specific target(s) and MoA or a library of 56 compounds belonging to 19 different classes of chemical compounds on A549 human lung adenocarcinoma cells. This experiment resulted in the publication of ProTargetMiner (http://protargetminer.genexplain.com), a curated, publicly available, expandable proteome signature library that represents a valuable tool in drug discovery.^181^ Ruprecht et al., by combining middle throughput label-free MS proteomics and biostatistics, characterized the molecular mechanisms of action of 50 drugs employed to treat lung cancer, including HDAC inhibitors, TK inhibitors, CDK inhibitors, and MAP2K1/2-MAPK1/3 inhibitors.^182^ Through PCA analysis and aggregation analysis of the differentially expressed proteins for each cell treatment, the authors mapped the biochemical networks modified by each drug class and defined their biochemical fingerprint around the specific target.^182^ MS proteomic data also led the team to discover that inhibition of mitochondrial function is an off-target mechanism of the MAP2K1/2 inhibitor **PD184352**, and that the ALK inhibitor ceritinib modulates autophagy.^182^ Still using MS proteomics, El-Baba et al. identified 197 proteins affected by **ST1926** administration, a synthetic retinoid used to treat glioblastoma multiforme (GBM), which targets POLA1, revealing its broad impact on key cancer pathways.^183^ The investigators discovered that **ST1926** treatment significantly reduces POLA1 levels, leading to cell cycle arrest, DNA damage, and apoptosis in GBM cells.^183^ An innovative, high-throughput platform for the cellular screening of drug phenotypes by dose-resolved expression proteomics has lately been introduced by Eckert et al.^184^ The DecryptE platform enhanced the understanding of the MoA of drugs, focusing on the effects of 144 drugs and research compounds across 8000 proteins, by generating over 1 million dose–response curves of protein expression.^184^ The authors discovered that the proteome modulation is strictly dependent not only on their MoA but also on the concentration used, i.e., some drugs, such as HDAC inhibitors, affect numerous proteins, while others, like certain kinase inhibitors, cause more specific changes.^184^ All of these case studies exemplify how **MS proteomics**, typically a label-free pipeline to accommodate more conditions, has been utilized to investigate the **MoA** of one or more compounds.

### 5.1 Hydrogen–Deuterium Exchange MS Proteomics

Hydrogen–deuterium exchange MS (HDX-MS) is a chemoproteomics technique that plays a central role in drug discovery by providing insights into protein structure, dynamics, and interactions with potential therapeutic agents, either small molecules or biopharmaceuticals.^185^ This technique allows investigators to characterize how proteins change conformation upon binding with ligands, revealing the main binding sites, strength, and the effects of various molecules on protein stability.^186^ By measuring the rate of deuterium incorporation into protein structures, HDX-MS can reveal the dynamics of protein–drug interactions, enabling the identification of allosteric or secondary binding sites, and help in the phase of drug optimization.^187^ Briefly, the protein or protein mixture previously purified is incubated with the investigated ligand(s) at different concentrations in the presence of D_2_O, to allow the protein’s mobile protons to exchange with deuterium.^187^ It is assumed that a good ligand can screen the protein's protons close to its binding site and engage in protein–ligand interaction.^187^ The protein is then quickly digested into peptides, and the rate of deuterium incorporation is then measured by high-resolution MS, as reported in Figure 11.^188^

**Figure 11:**
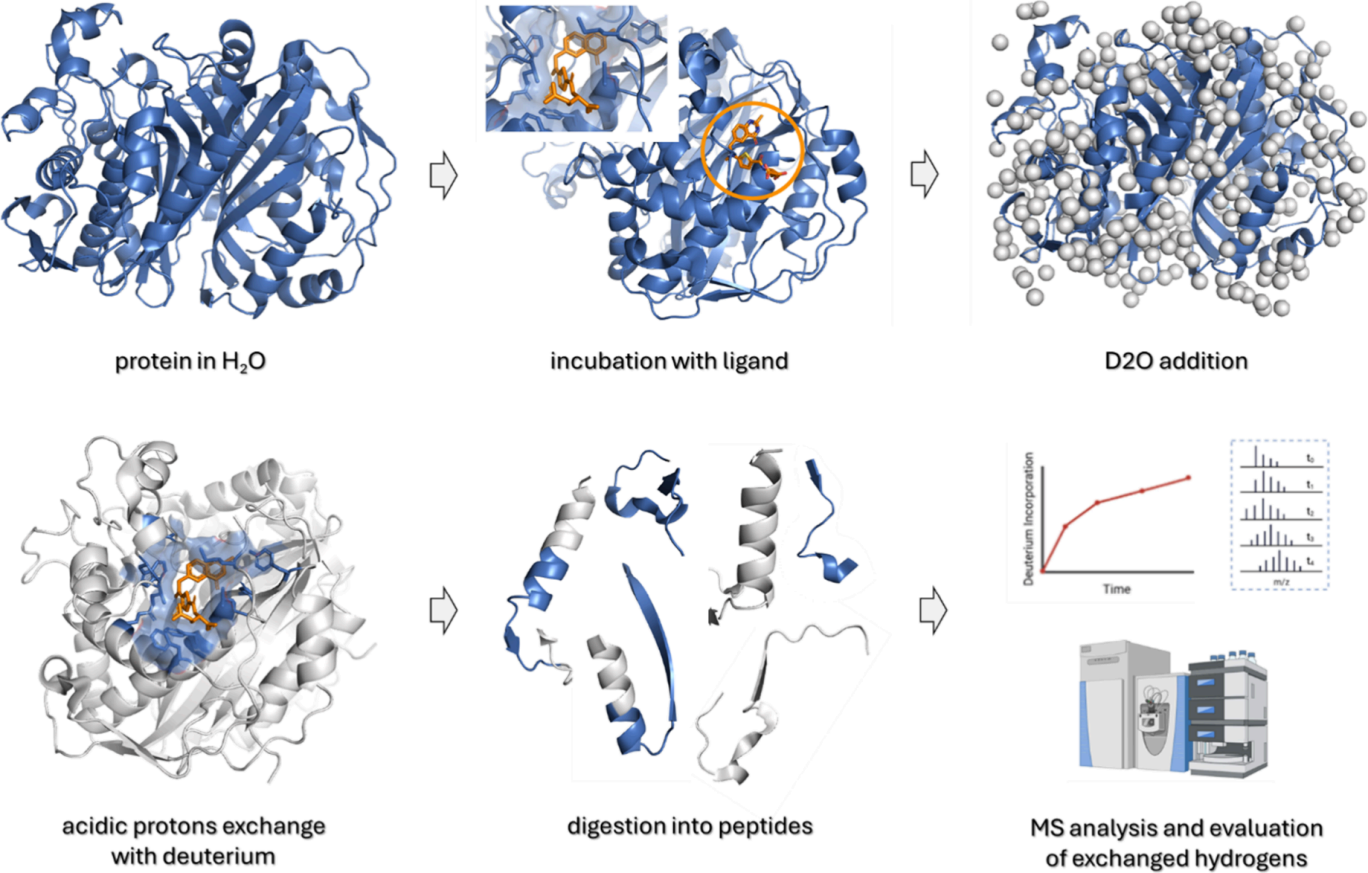
HDX experiment scheme. After ligand incubation, D2O is added for an established timelapse before protein digestion. And acidic pH quenches the reaction and unfolds the protein and analyzed with HRMS. HR instruments like TOF or Orbitrap can discriminate small hydrogen mass shifts, which makes it easy to identify which peptides and single amino acid underwent major deuterium incorporation. In the present figure, the protein with its hydrogens is represented in blue cartoon, D_2_O is represented as gray spheres and protein residues that have incorporated deuterium as gray cartoons. Thymidylate synthase in complex with methotrexate (PDB:5X66) was used to generate this image on BioRender.

The ability to analyze also large complexes and challenging targets, such as membrane proteins and disordered regions, makes HDX-MS, particularly valuable in the development of antibodies.^189^ Furthermore, advancements in tools and data processing for HDX-MS experimental interpretation have enhanced the efficiency and accuracy of HDX-MS.^190^ Despite being like the technology of the previously described PAL, HDX-MS has the advantage of needing no chemistry or probe design, making it ideal to investigate lead compounds with their original structure and no modifications.^191^ Also, correlating the HDX-MS profile of large numbers of ligands during the hit optimization phase with their functional outputs enables the development of SARs and the delineation of hit scaffold classes based on functional selectivity.^192^ Ow et al. exploited this technique to explore the novel binding site of garadacimab (**CSL312**) on the Hageman factor (FXII), an essential component in the intrinsic coagulation cascade and a therapeutic target for the prophylactic treatment of hereditary angioedema.^193^ The authors revealed an additional “side pocket,” complementarity-determining regions (CDRs) close to the antibody main recognition site, which can be exploited as a potential paratope for the development of new, more selective FXII inhibitors.^193^ Similarly, Woods et al. investigated the tyrosine phosphatase 1B (PTP1B) structure, a validated therapeutic target for obesity, diabetes, and certain types of solid cancer.^194^ In particular, by HDX-MS, they mapped a particular rigid backbone sequence involved in the binding with the active-site inhibitor **TCS401**, which may serve for orthosteric compound design and optimization. Also, they mapped allosteric binding sites of PTP1B for small-molecule inhibitors.^194^ Another example of HDX-MS-driven compound optimization is represented by the study of Song et al. on SARS-CoV-2 3-chymotrypsin-like proteases (3CLpro or M^pro^), through which an allosterically regulated dimerization of this protein that affects its activity was characterized. Based on the HDX-MS data, the authors propose the scaffold of gastrodenol as a lead compound with dissociative activity on 3CPLpro for further investigation.^194^ In a similar article about another viral target, the activity of lofanarib in inhibiting fusion protein was investigated to treat the early stages of respiratory syncytial virus infection. The time-dependent HDX-MS assay demonstrated that lofanarib interacts with 10 key residues of the fusion protein (EC_50_ 57.7 ± 15.4 nM in HEp-2 cells) to block its conformational exchange that triggers viral fusion and puts the basis to develop more specific inhibitors from this lead compound.^194^ Overall, the literature demonstrates that HDX-MS provides several key advantages over a wide range of conventional techniques used to identify ligand-binding sites, including X-ray crystallography, cryoelectron microscopy (cryo-EM), nuclear magnetic resonance (NMR), surface plasmon resonance (SPR), and differential scanning fluorimetry (DSF). Unlike crystallography or cryo-EM, which require stable, purified, and often crystallizable proteins, HDX-MS operates in solution under near-physiological conditions and can probe dynamic, flexible, or disordered regions that are invisible in static structural models. Compared with NMR, HDX-MS is faster, is compatible with larger proteins and complexes, and requires much lower sample quantities. While SPR and DSF report on global binding events or thermal stability changes, HDX-MS uniquely provides site-resolved information by detecting changes in the backbone amide hydrogen exchange upon ligand binding. This enables the precise localization of binding regions, the identification of allosteric effects, and the characterization of conformational dynamics across the entire protein. Overall, HDX-MS delivers a powerful combination of structural sensitivity, throughput, and applicability, making it superior for mapping ligand-binding sites in complex protein systems.

### 5.2 Co-Immunoprecipitation Followed by MS (Co-IP-MS)

Co-IP-MS is an MS proteomics technique used to study protein–protein interactions (PPIs) by combining Co-IP with MS proteomics.^195^ The main purposes of Co-IP-MS are the mapping of protein interaction networks (PINs) within a cell or biological fluid in a quali-quantitative network, often before and after administration of a specific compound, or in the presence and absence of a metabolic disease, which makes it a gold standard technique to investigate the efficacy of protein:protein inhibitor candidates.^196^ The considered cells or tissues, properly treated, are broken open under specific conditions that preserve protein interactions, e.g., mechanical lysis over chemical detergents.^197^ A specific antibody targeting the protein of interest, known as the “bait” protein, is then introduced in the system, which is usually bound to a support, such as Protein A/G beads, through a Schiff’s base.^198^ This helps isolate the bait protein along with any associated binding partners (“prey” proteins).^199^ After incubation, unbound proteins and other cellular components are washed away with a specific buffered solution, leaving only the bait protein and its prey attached to the beads.^200^ Typically, low-salt or high-salt conditions are used to help disrupt specific interactions, along with detergents like Triton X-100 or NP-40 and reducing agents. The isolated protein complex is then subjected to a standard bottom-up MS proteomics workflow, and from the composition of the quantified peptides, it is possible to determine which proteins interact with the bait protein under determined conditions.^201^ Sometimes, cell lysate PPIs can be stabilized through chemical cross-linking, e.g., DSP (dithiobis-succinimidyl propionate) to allow the identification of even short-lived complexes.^202^ In this case, the covalent modification needs to be reverted with mild reducing agents like TCEP before MS analysis.^203^ Figure 12 reports a schematic workflow for bead immobilization (Figure 13A) and affinity capture (Figure 13B). This was the case of Liu et al., who demonstrated the antinflammatory activity of isoliquiritigenin (ISL) by combining data from RT-qPCR and Co-IP-MS experiments and proving that ISL covalently binds to MS2 and disrupts the formation of the LPS/MD2/toll-like receptor 4 complex, thus resulting in significantly alleviated lung injury in LPS-induced mice.^204^ Following a similar experimental pipeline, Zhihuai et al. investigated the pharmacological activity of liquidambaric acid (i.e., betulonic acid, or HY-N1451-31) in the disruption of STAMBPL1/NRF2 complex, which would promote DUB activity and eliminate ubiquitin molecules attached to NRF2, thus protecting it from proteasome-mediated degradation.^205^ Several years before, Gorini et al. succeeded in Co-IP experiments with dynamin-1 coprecipitating BKCa channels from native mouse brain preparations.^206^ This interaction was reproducibly detected by Western blotting, confirming the association between these two proteins and suggesting a specific and stable interaction under physiological conditions, pointing toward a functional relationship between dynamin-1 and BKCa channel complexes in neuronal tissue.^206^ In their works, Hossain and colleagues also integrated Co-IP-MS in their innovative mathematical-based drug discovery decision-tree workflow, which combines a novel MS proteomics assay and a mathematical PK/PD model specifically designed for covalent drugs. Indeed, the model was validated for two approved drugs and proposed for a class of promising ALS drug candidates targeting SOD1.^206^

**Figure 12:**
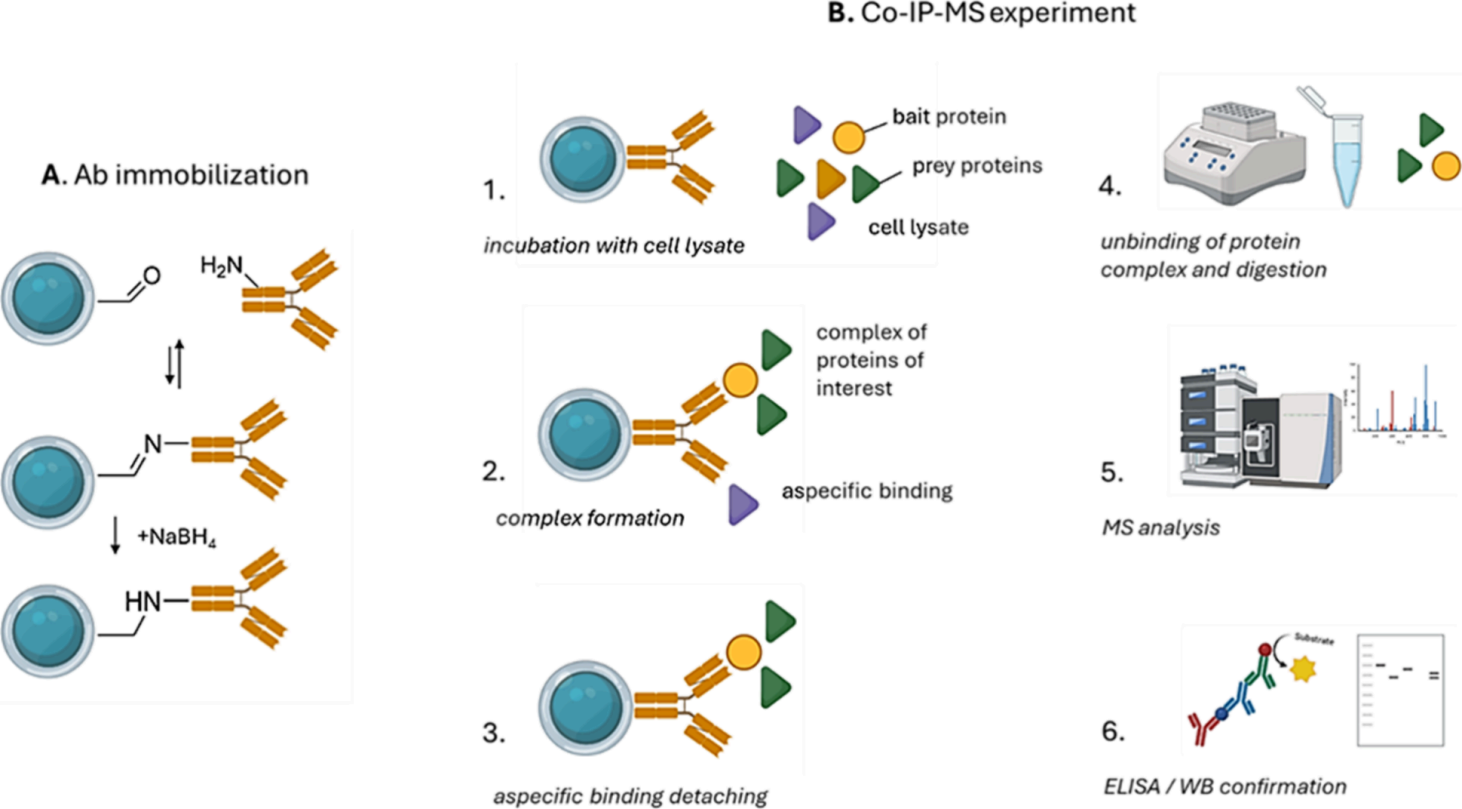
Schematization of a Co-immunoprecipitation-MS experiment. (A) Appropriate antibody toward bait protein is incubated with agarose beads functionalized with aldehyde-carrying ketones, which reversibly react with lysins of the antibodies by giving a Schiff’s base. The formed imine is reduced by NaBH_4_ to stabilize the bond. (B) Agarose beads properly functionalized with antibodies are incubated with cell lysate (1) to form the complex between protein of interest and prey proteins, including nonspecific binding (2). Nonspecific bound proteins are detached by varying salt or pH concentration (3), then the formed complex is detached and digested with a standard bottom-up MS procedure (4). Obtained peptides are analyzed with LC–MS/MS (5), and an orthogonal analysis with either ELISA, WB, or other immunohistochemical techniques is performed (6). The image was created with BiorRender.

**Figure 13:**
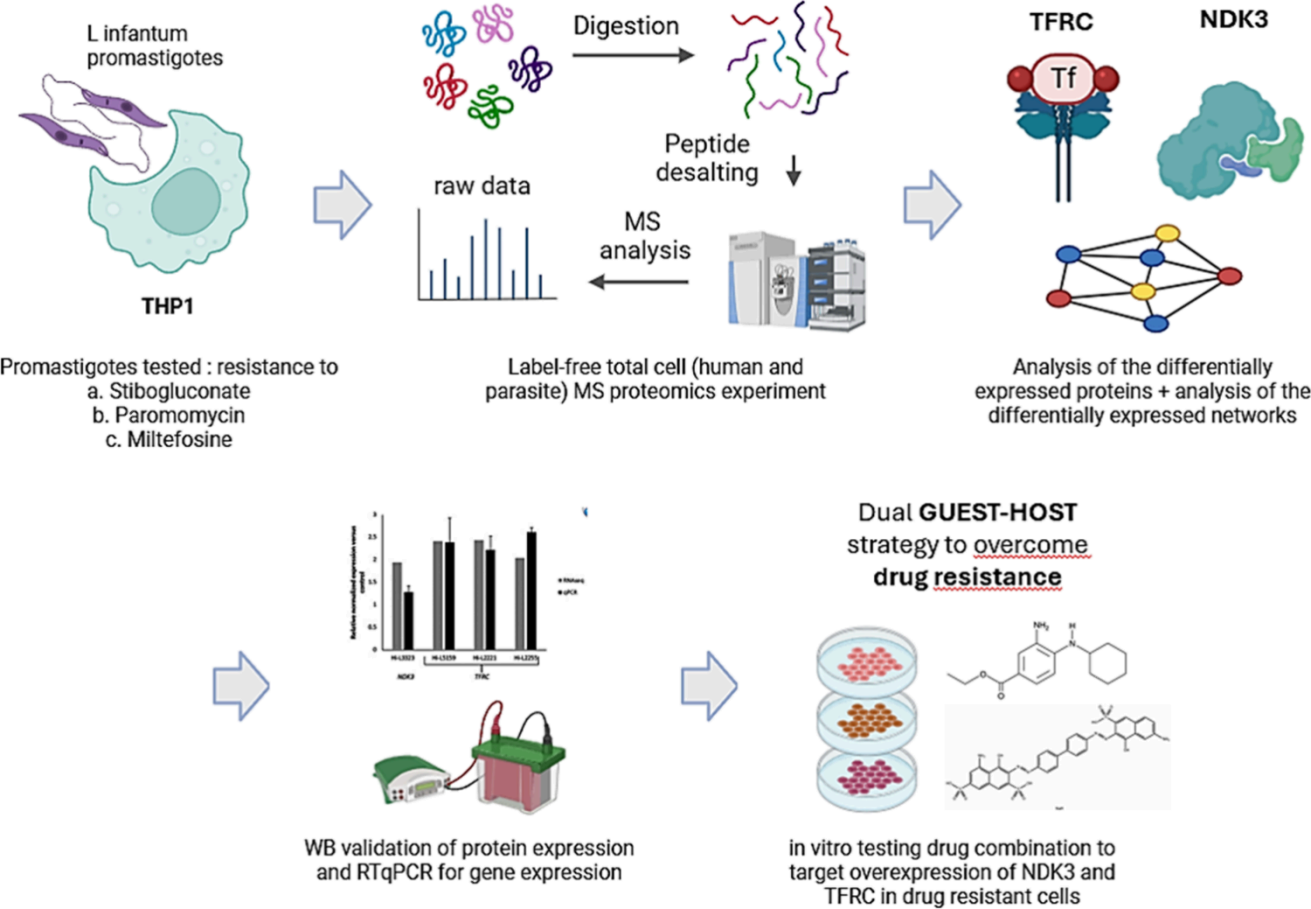
Examples of MS proteomics used for the characterization of drug resistance patterns in *L. infantum*. Tagliazucchi and colleagues exposed three drug-resistant *L. infantum* strains from clinical isolates to THP-1 macrophages (human). The system in acute infection was submitted to total MS proteomics. Human proteins were distinguished from the parasitic ones and quantified. NDK3 and TFRC were marked as overexpressed in miltefosine and paromomycin strains with respect to nonresistant *L. infantum* strains. The authors validate their findings through WB and quantitation of NDK3 and TFRC transcripts by RT-qPCR. As the authors’ hypothesis includes NDK3 and TFRC being upregulated in host cells by the guest for iron and nucleoside metabolic supply, contributing to drug resistance, ongoing experiments include the administration of NDK3 and TFRC inhibitors in a perspective to also reduce the resistance index.^214^ The figure was realized with BioRender and is based on the content of the referenced publication.

Overall, the preference of MS-guided Co-IP offers distinct advantages within the drug discovery pipeline, particularly when studying target engagement and MoA, compared with other common approaches such as surface plasmon resonance (SPR), cellular thermal shift assays (CETSA), or differential scanning fluorimetry (DSF). Unlike biophysical assays such as SPR or DSF, which rely on purified recombinant proteins and primarily measure direct ligand–target binding in vitro, Co-IP-MS provides information within a cellular or native physiological context. This allows the identification of drug–target interactions as they occur in living systems, preserving their natural conformation. As a result, Co-IP-MS offers a more realistic view of target engagement, especially for compounds acting within multiprotein assemblies or signaling complexes. Another major advantage of Co-IP-MS is its ability to capture both direct and indirect effects of compound binding. While thermal stability-based methods such as CETSA detect changes in protein folding upon ligand binding, they cannot directly reveal how those interactions affect the broader interactome. In contrast, Co-IP-MS can detect changes in the composition or abundance of protein complexes following drug treatment, highlighting secondary binding partners or interaction networks that are stabilized, disrupted, or newly formed. This is particularly valuable for drugs that act allosterically, modulate scaffolding proteins. Furthermore, Co-IP-MS offers unbiased identification of interacting partners without prior assumptions about the molecular mechanism, making it a powerful discovery tool for elucidating mechanisms of action of novel compounds or phenotypic hits. When compared with label-based affinity methods such as DARTS, Co-IP-MS has the advantage of relying on endogenous PPIs rather than on chemical derivatization of the ligand, which can sometimes alter binding properties.

## 6 MS Proteomics for the Characterization of Drug Resistance Mechanisms and New Drug Combinations

MS proteomics is increasingly recognized as a powerful and accurate tool for characterizing drug resistance in various diseases, particularly cancer, bacterial diseases, and parasitic infections.^207^ By analyzing the proteomic profiles of resistant and susceptible cell lines, tissues, or clinical isolates, or by recreating in vitro certain conditions to induce a resistant phenotype, it is possible to identify specific proteins and map differentially regulated pathways that associated with or cause resistance mechanisms.^16,208^ This allows to understand key proteins responsible for therapeutic failure, to improve compound optimization, and to study possible drug combination to escape or delay the onset of resistance.^209,210^ For instance, MS Proteomics evidenced in HER2+ breast cancer 3D cell models (approximately 15–30% exhibit HER2 amplification, triggering enhanced signaling through various pathways, notably the PI3K/Akt and MAPK pathways, which promote cell proliferation and survival), the MS proteomic characterization revealed the involvement of mitochondrial complex I in acquired resistance to trastuzumab, highlighting how metabolic pathway modulation can influence treatment outcomes.^211^ This could provide personalized therapeutic strategies, increasing the efficacy of drug treatment while reducing its side effects.^211^ HDAC inhibitors represent important drugs for cancer therapy, but the unclear resistance mechanisms greatly limit their clinical applications.^212^ The contribution of Hao et al., who exploits the (TMT)-based MS proteomics approach on HDACi-sensitive and -resistant cell lines, identifies a set of proteins responsible for HDAC-resistant phenotype (including MRTO4, PES1, WDR74, and NOP16), and proposes a combination strategy that targets both HDAC and one of the main causes of HDCA mutations to delay the onset of drug resistance during chemotherapy.^212^

Additionally, label-free MS proteomics methods have been employed to analyze the global proteome of drug-resistant bacteria, such as *Acinetobacter baumannii,* revealing changes in key virulence proteins that contribute to antibiotic resistance, such as the membrane efflux pumps AdeB and AdeDE, which actively expel a wide range of antibiotics, contributing to its multidrug-resistant (MDR) phenotype. This approach allows for the identification of proteins that may serve as biomarkers for resistance or potential therapeutic targets.^15^ A very similar pipeline was used by Abril et al. to do a global shotgun proteomics characterization for pathogenic *Listeria* species, particularly *L. monocytogenes*, and describe their phenotypes of antibiotic resistance.^213^ The study identified proteins associated with beta-lactamase activity, penicillin-binding proteins, and other resistance mechanisms to antibiotics such as gentamicin, kanamycin, fosfomycin, and tetracycline. Proteins like internalins (InlA, InlB), which are involved in bacterial entry into host cells, and peptidases that aid in host invasion were highlighted, were mapped as key player of the phenotype resistance, and the Lactococcin 972 family was proposed as new target for a drug combination approach.^213^ MS Proteomics is also employed to gain insights into parasitic chemoresistance mechanisms. It is the case, for instance, of Saboia-Vahia et al., that compared the protein expression profiles of a miltefosine-resistant *L. infantum* strain with that of the wild-type (WT) strain.^214^ They experimentally induced resistance in the parasites through six months of continuous exposure to increasing doses of miltefosine and identified proteins like ABC transporters, sterol biosynthesis enzymes and phospholipid transporting ATP-ases as potentially involved in the resistant phenotype.^214^ Similar results but on THP-1 infecting promastigotes were obtained by Tagliazucchi et al., who identified an overexpression of energetic (TCA) metabolism, fatty acid biosynthetic proteins and membrane lipid metabolism in miltefosine and antimonial resistant *L. infantum* clinical isolates (Figure 13).^215^

Only recent literature discloses the potentialities of MS proteomics and kinomics in identifying effective drug combinations compared with more conventional molecular and cellular techniques such as genomics, transcriptomics, WB, or immunoassays. Unlike genomic or transcriptomic profiling, which reveals only potential regulatory changes (i.e., on a genic base), MS proteomics reveals true functional effectors of drug response. This allows the overall characterization of adaptive signaling rewiring, compensatory pathway activation, and metabolic remodeling that underlie resistance phenotypes. In contrast to targeted techniques like WB, ELISA, or flow cytometry, which are limited to predefined proteins and require specific antibodies, MS proteomics provides a system-wide coverage without prior knowledge of targets (i.e., off-target information), and phosphoproteomics can uncover alterations in kinase signaling networks driving resistance. Moreover, by integrating whole system proteomic with phosphoproteomic data, MS enables rational design of synergistic drug combinations that cotarget reactivated or compensatory pathways, an insight often missed by single-*omic* or targeted assays. Overall, the purposes of MS proteomics in drug-resistant studies can be summarized in Table 6.

**Table 6:** Summary of MS Proteomics in Drug-Resistant Studies, with Their Related Outcomes and the Potential Outcomes within Medicinal Chemistry Programs

general concept	actual outcomes	potential achievements
use of resistant vs sensitive models (cell lines, tissues, bacterial/parasitic/viral isolates) to compare global proteomic profiles	identification of key differentially expressed proteins	discovery of novel therapeutic targets
	mapping of deregulated pathways	identification of existing inhibitors to block or modulate dysregulated pathways
	functional validation, *i.e*., metadata confirmation, orthogonal validation (e.g., WB, ELISA)	experimental administration and testing molecules on resistant cells/isolates
induction of resistance in vitro through prolonged drug exposure and subsequent proteomic comparison	generation of resistant strains/models	prediction of clinical resistance evolution
	discovery of resistance mechanisms	development of early intervention therapies
		biomarker discovery for resistance monitoring
host–pathogen interaction proteomics to identify guest-induced changes in host cells	separation and quantification of host vs parasite proteome	targeting host cell pathways hijacked by parasites
	identification of guest-induced host reprogramming to sustain guest survival	dual targeting (host + pathogen) strategies
		potential reduction of resistance development

## 7 MS Proteomics in Toxicology and Adverse Outcome Pathways Identification

One of the modern approaches that has changed toxicology and safety assessment in drug discovery is the adverse outcome pathway (AOP) framework.^216^ AOPs provide a structured way of connecting molecular-level interactions caused by chemicals (including drugs) to adverse health outcomes.^217^ AOPs facilitate the prediction of potential hazards at the early stages of drug development, helping to reduce late-stage failures in clinical trials due to safety concerns.^218,219^ An AOP is a conceptual framework that describes the progression from a molecular-initiating event (MIE), which is the first interaction between a drug or other chemical and a biological target, through a series of intermediate steps, ultimately leading to an adverse outcome at the individual or population level.^220,221^ These steps form a cause-effect chain, linking biological mechanisms to phenotypical effects that can be measured and monitored.^222^ A typical AOP consists of the following elements: i. an MIE), i.e., a direct interaction between a chemical and a biological molecule (e.g., a drug binding to a receptor or an enzyme); ii. the key events (KEs), which encompass the biologically meaningful changes that occur at the cellular, tissue, or organ level following the MIE (these are measurable processes such as changes in gene expression, proteomic changes, cellular stress, or inflammation), and finally, iii. the adverse outcome (AO), that is, the ultimate harmful biological effect, which could be organ toxicity, impaired reproduction, developmental defects, or death.^220,223^ The incorporation of AOPs into drug discovery offers multiple advantages. Mainly, AOPs help in understanding how a drug’s molecular interactions lead to toxicity, providing mechanistic insights into potential safety issues early in the discovery phase.^224^ This allows to design compounds that avoid toxic pathways. AOP studies also help in the reduction in animal testing. Since AOPs are based on mechanistic understanding, they can be used in combination with alternative testing methods, such as in vitro models and computational simulations.^225^ AOPs facilitate the prediction of potential toxicological outcomes based on early molecular events.^2^ By screening compounds for interactions with key molecular targets, it is possible to predict whether a drug candidate is likely to cause an adverse outcome before it progresses into animal or human testing.^2^ The AOP framework is compatible with data from various sources, including in vitro assays, omics technologies (e.g., genomics, proteomics), and computational models.^226^ This integration enables a more comprehensive assessment of a drug’s safety, incorporating different layers of biological data into the decision-making process.^216^ For instance, Bakker et al. recently investigated the cross-integration of transcriptomic and proteomic data to improve the AOP context by focusing on *Folsomia candida*, a soil invertebrate, exposed to imidacloprid, which overstimulates the nicotinic acetylcholine receptor (nAChR), leading to neuronal disruption.^227^ By collecting data at 12 h intervals up to 72 h, the researchers identified the most significant molecular changes at the 48-h mark. This time point was crucial for observing differential protein expression patterns, particularly in pathways related to neurodegeneration and synaptic signaling.^227^ Another case study of MS proteomics in AOPs characterization was well described by Liu et al., who discusses the effects of polystyrene nanoplastics on *Daphnia pulex*, a key species in aquatic ecosystems.^228^ The group explored how nanoplastics, particularly spherical polystyrene, affect reproduction and growth in *Daphnia*. Using MS proteomics, the researchers identified changes in protein expression related to oxidative stress, energy metabolism, signaling pathways (such as mTOR and Jak-STAT), and cuticle development. This outcome allowed for refinement of the AOP for nAChR activation by adding new key events, such as GABA signaling and mitochondrial dysfunction. These insights also help identify biomarkers for imidacloprid exposure, contributing to more efficient environmental risk assessment.^228^ Table 7 depicts the potentialities and limitations of MS proteomics studies of AOP events.

**Table 7:** Potentiality of MS Proteomics Studies of AOP Events and Their Limitations in the Context of Drug Discovery

potential	limitations
high sensitivity and specificity: MS proteomics can detect and quantify low-abundance proteins, allowing the identification of early molecular changes in response to toxicants	**sample complexity and dynamic range**: biological samples often contain proteins with a wide range of abundances. High-abundance proteins (HAPs), like albumin in serum, can mask the detection of low-abundance proteins (LAPs), potentially overlooking critical components of AOPs
global proteome coverage: advanced MS techniques, such as shotgun proteomics, allow for the broad profiling of proteins, facilitating the mapping of entire signaling pathways involved in AOPs	**data analysis challenges**: the vast amount of data generated requires sophisticated bioinformatics tools and expertise. Misinterpretation can occur due to issues like peptide degeneracy and incomplete databases.
quantitative analysis: label-free and label-based quantification methods enable the measurement of protein expression changes over time, aiding in the temporal mapping of AOPs.	**ion suppression effects**: co-eluting compounds from complex matrices can suppress ionization efficiency, leading to inaccurate quantification and potential false negatives. This can be overcome only if standard reference compounds are available.
detection of PTMs: MS is adept at identifying PTMs, such as phosphorylation and oxidation, which are crucial in signaling events leading to AOPs	**incomplete PTM coverage**: some PTMs are labile or occur at low stoichiometry, making them difficult to detect and quantify accurately.

## 8 MS Proteomics for Biomarker Discovery, Verification, and Validation

Biomarkers are defined as biological molecules that indicate physiological or pathological states, playing a vital role in disease diagnosis, prognosis, and therapeutic monitoring.^229^ MS proteomics has become a powerful tool also in accelerating the discovery pipeline and diminishing the costs of biomarker studies, due to its sensitivity and ability to analyze complex biological samples.^230^ Biomarker discovery using MS-based proteomics involves a multiphase process that can be divided in three main phases: discovery, verification, and validation.^231^ Each phase leverages different high-throughput MS technologies to explore the proteomic landscape and identify potential biomarkers, combined with orthogonal techniques.^175^ In the discovery phase, MS-based proteomics is usually used to compare protein expression in biological samples (e.g., blood, tissue, and urine) from diseased and healthy individuals. This phase is performed on disease groups compared to control groups and often involve a validation of the corresponding gene expression levels.^232^ Following discovery, candidate biomarkers (<10) undergo verification stage. Targeted MS approaches such as selected reaction monitoring (SRM) or parallel reaction monitoring (PRM) have extensively been used for this purpose and have replaced older optical techniques and immunoassays.^32,233^ These techniques allow for the quantification of specific proteins with high precision and sensitivity but need to be combined with orthogonal assays including immunohistochemistry (ELISA, WB, etc) and kinetic assays, with the purpose of assessing sensitivity.^234^ The final stage, validation, involves testing biomarkers in larger, independent cohorts to assess their clinical utility.^235^ This phase often employs multiplexed immunohistochemistry assays, or MS to quantify several biomarkers simultaneously, ensuring the robustness of findings.^236^ Finally, when biomarker is validated on a larger scale (*n* > 1000 patients), data can be submitted to national and supranational health authorities for revision.^237^ A general scheme of the biomarker discovery process with MS proteomics integration is illustrated in Figure 14.

**Figure 14:**
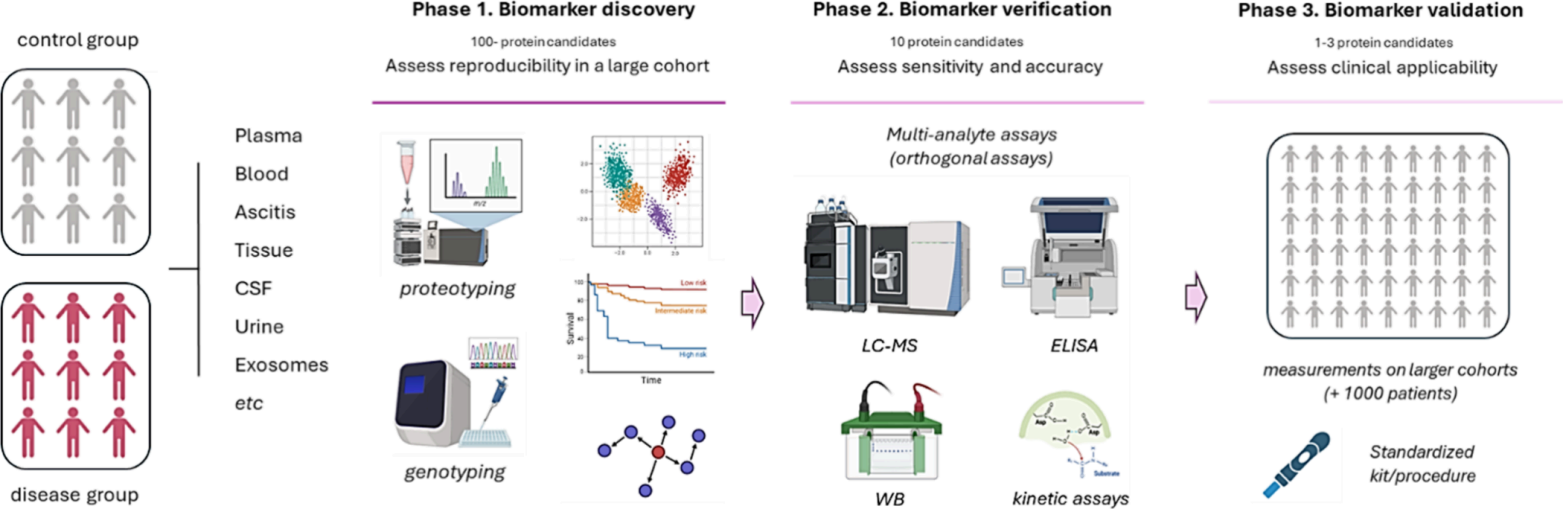
General protein biomarker discovery and validation pipeline. Phase 1 starts with the hypothesis generation of the involvement of a group of proteins/pathways in the progression of a disease. The total proteome from different biological samples (e.g., blood, urine, CSF) of the disease group is measured and compared to that of the control group. Multiparametric analysis (i.e., ANOVA) is then applied to verify which proteins/networks are significantly differentially regulated in the disease group. If possible, a primary validation of proteins is performed on their transcripts to ensure that the protein’s differential expression has a genetic basis. Phase 1 assesses reproducibility in large cohorts and represents an explorative stage. Phase 2 restricts protein candidates *n* < 10, and consists of multianalyte assays with different techniques, including targeted MS, immunohistochemical assays or enzymatic/colorimetric detection. The aim of phase 2 is to assess sensitivity with different techniques and their accuracy. Phase 3, i.e. biomarker validation, is performed on larger cohorts (>1000 patients), in randomized, double-blind trials to assess clinical applicability. In this phase, the technique of standard measurement is chosen, which is often robust, poorly affected by false negatives, and affordable on a large scale (often kits are produced if the procedure is noninvasive). At this point, the biomarker data are submitted to health authorities for revision and qualification. The image was created with BioRender.

A notable platform used for early phase biomarkers investigation was The Clinical Proteomic Tumor Analysis Consortium (CPTAC), who, led by the National Cancer Institute (NCI), has integrated proteomic data with genomic and transcriptomic information from The Cancer Genome Atlas (TCGA), by giving birth to the so-called proteogenomic field.^238^ The consortium uses advanced MS proteomics and other proteomic technologies to produce deep proteomic data sets linked to genomic data from projects like TCGA, facilitating a unified understanding of cancer biology and enabling the development of targeted cancer treatments.^239^ A schematic pipeline of CPTAC is represented in Figure 15A.

**Figure 15:**
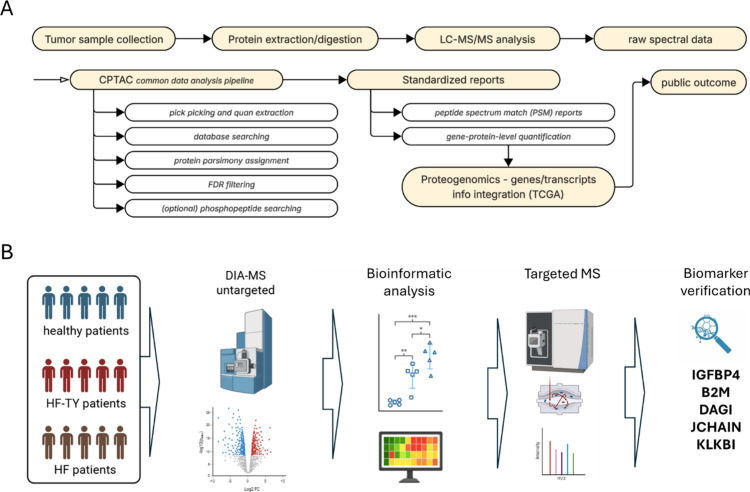
(A) Schematic representation of the CPTAC operative workflow, from sample collection to data repository. (B) Workflow representation of the whole serum MS proteomics experiment by Lan et al. to investigate potential biomarkers for heart failure patients with phlegm-blood stasis syndrome. A set of 84 DEPs with a DIA-untargeted experiment, 37 of which were chosen for a targeted analysis with the PRM method, and five validated proteins with high sensitivity and specificity, including insulin-like growth factor-binding protein 4 (IGFBP4), β-2-microglobulin (B2M), dystroglycan (DAG1), immunoglobulin J chain (JCHAIN), and kallikrein B1 (KLKB1). The image was created with BioRender.

For example, a proteogenomic study assisted by the CPTAC helped identify S100A9 and GRN as potential combinatorial biomarkers for the early detection of hepatocellular carcinoma (HCC) from urine samples, offering two noninvasive diagnostic tools.^240,241^ In lung adenocarcinoma, fusion proteins such as EML4-ALK and HMBOX1-ALK, discovered through RNA-seq and proteomics integration, have been identified as promising biomarkers, enhancing the understanding of tumor-specific mutations and guiding targeted therapies.^242^ Similarly, in amyotrophic lateral sclerosis (ALS), ultrasensitive MS assays have been developed to measure isoform levels of C9ORF72, a mutation closely associated with FTD and ALS.^243^ In C9ORF72 mutation carriers, the long isoform was found to be significantly reduced in brain tissue.^244^ A recent study by Åkesson et al. utilized a proximity-extension MS proteomics assay combined with next-generation sequencing (Olink Explore HT^245^) to quantify 1,463 proteins in CSF and plasma from patients with early-stage multiple sclerosis.^230^ Lower levels of neurofilament light chain (NfL) in CSF were found to be effective in predicting the absence of disease activity two years after sampling (AUC of survival rate = 0.77), and a combination of 11 proteins from CSF (e.g., CXCL13, LTA, NfL) were able to accurately predict the severity of disability worsening (AUC of survival rate = 0.90).^230^ Another high-relevance exploratory study comes from Zertuche-Martínez et al., who employed label-free MS proteomics to discover candidate biomarkers from plasma-derived extracellular vesicles (EVs) of patients with cirrhosis and HCC.^246^ EVs represent an attractive source of biomarkers due to their biomolecular cargo, and among the total 248 identified proteins, 5 DEPs were found relevant to cirrhosis, and 12 DEPs to HCC, with four (LCAT, SERPINF2, A2M, CRP) of both interest that should be investigated further.^246^ In a whole-serum MS proteomics experiment, Lan et al. investigated potential biomarkers for heart failure patients with phlegm-blood stasis syndrome, by comparing the circulating proteome of 20 healthy and 40 heart failure patients.^247^ They obtained a set of 84 DEPs with a DIA-untargeted experiment, 37 of which were chosen for a targeted analysis with the PRM method, and five validated proteins with high sensitivity and specificity, including insulin-like growth factor-binding protein 4 (IGFBP4), β-2-microglobulin (B2M), dystroglycan (DAG1), immunoglobulin J chain (JCHAIN), and kallikrein B1 (KLKB1), were considered potential biomarkers for heart failure patients with phlegm-blood stasis syndrome.^247^ Their experimental design is represented in Figure 15B. The same untargeted MS proteomics-bioinformatic-targeted MS proteomics pipeline was exploited to validate putative biomarkers from a data set of proteins coming from an explorative analysis by Sanni et al. for the identification of candidate serum biomarkers in patients with narcolepsy Type 1.^248^ Here the authors investigated 14 of the DEPs identified from the untargeted analysis, and eight of them maintained the same trend of fold change in the PRM analysis. Inter-α-inhibitor heavy chain 4 (ITIH4), fibronectin 1 (FN1), and complement component (C5) were proposed for further studies after cellular localization analysis.^248^

The recent research from Tapia et al. about HER2+ breast cancer (BR) resistance phenotype is a good example of the target verification stage.^211^ Indeed, the authors compared trastuzumab-responding spheroids and trastuzumab-resistant spheroids and found that resistant 3D cells displayed a downregulation of carbohydrate metabolism and upregulation of mitochondria organization proteins, the tricarboxylic acid cycle, and oxidative phosphorylation.^211^ A further biostatistical analysis demonstrated that up regulation of Complex I proteins NDUFA10 and NDUFS2 was associated with high clinical risk and worse survival for HER2+ BC patients, which was confirmed by a retrospective analysis of clinical metadata on mRNA expression of mitochondrial Complex I genes in BR patients.^211^ Still exploring the field of target verification, researchers recently used a targeted proteomics approach to analyze plasma samples from individuals with early-stage Parkinson’s disease, premotor individuals with isolated REM sleep behavior disorder (iRBD), and healthy controls.^231^ Proteotypic peptides to be monitored were chosen from previous exploratory untargeted studies, like in Hällqvist et al.,^249^ and from known literature data on Parkinson’s and neural aging. Eight proteins, including granulin precursor, mannan-binding-lectin-serine-peptidase-2, and plasma-protease-C1-inhibitor, were identified in the targeted experiment as strong predictors for Parkinson’s, and machine learning models classified 79% of premotor individuals as at risk for PD up to 7 years before motor symptoms, with 100% accuracy in distinguishing Parkinson’s patients from healthy controls. This panel of blood biomarkers holds promise for early detection of Parkinson’s and could aid in selecting participants for clinical trials aimed at preventing or delaying the onset of motor symptoms.^249^

Since secretome encompasses signaling molecules, cytokines, and growth factors, which play key roles in intercellular communication and disease progression, its analysis provides direct insights into drug mechanisms of action and novel biomarker identification.^250,251^ Closely related to MS secretomics is the analysis of EVs, including exosomes, which are nanosized vesicles released by virtually all cell types.^252^ Exosomes, the main components of secretome, carry a diverse cargo of proteins, nucleic acids and lipids, reflecting the physiological or pathological state of their cell of origin.^253^ Their proteome is enriched in membrane proteins, signaling mediators, and enzymes, many of which represent potential drug targets or biomarkers.^254^ MS-based exosome proteomics typically involves isolation of vesicles from plasma, CSF, or urine using ultracentrifugation, size-exclusion chromatography, or affinity capture, followed by traditional bottom-up proteomic analysis.^255^ Importantly, exosomes protect their cargo from degradation, making them a stable and rich source of novel biomarkers for pathology progression or ongoing therapy success. Many authors have studied these innovative sources of biomarkers coming from different biological tissues. This is the case of Li and colleagues, for instance, who profiled plasma circulating biomarkers in several cohorts of patients affected by lung and brain tumors and found that SELL and MUC5B protein levels could be used as diagnostic markers of brain tumor metastasis, while APOH, CD81, and CCT5 could help diagnose lung metastases in NSCLC.^256^ In a similar manner but for a different disease, Zhang et al. used MS proteomics of CSF exosomes to profile the disease activity and long-term prognosis in patients with multiple sclerosis. They considered a cohort of 143 patients vs 43 controls and found that NfL in CSF is superior in predicting the absence of disease activity two years after sampling. Also, they demonstrated that NfL, along with other 10 proteins (XCL13, LTA, FCN2, ICAM3, LY9, SLAMF7, TYMP, CHI3L1, FYB1, and TNFRSF1B) can constitute a panel of biomarkers that can predict disease worsening when corrected by normalized age-related severity score.^230^

Other successful examples demonstrate the potential of MS proteomics in biomarker discovery. Nevertheless, it remains a secondary technique for biomarker investigation, even though it is believed to have huge potential to become the technique of choice. Its main pros and cons are illustrated in Table 8.

**Table 8:** Pros and Cons of the Use of MS Proteomics over Other Techniques in the Biomarker Verification Process

feature	pros	cons
diagnostic accuracy	high specificity and sensitivity can enhance diagnostic power compared to traditional methods	some MS platforms lack robust validation in diverse clinical populations
multiplexing capability	MS allows simultaneous quantification of multiple biomarkers from a single sample	interpretation of multiplex data can be complex and less transparent for clinicians
noninvasive sample use	compatible with various biofluids (*e.g.,* plasma, urine, saliva), supporting noninvasive testing	variability in sample collection methods and processing can affect reproducibility
speed and throughput	automation and high-throughput workflows can accelerate clinical decision-making	workflow is still slower than some immunoassay-based platforms in point-of-care settings
standardization potential	emerging protocols are improving interlaboratory reproducibility. Need to uniform SOPs (standard operating procedures)	lack of universally accepted clinical standards and reference materials for MS biomarkers
regulatory support	targeted MS assays (like MRM/SRM) are increasingly accepted in regulatory submissions. Nontargeted methods remain unaccepted due to variability problems associated with normalization methods	full validation and official approval are time-consuming and require expensive clinical trials with respect to other quantification techniques
cost-effectiveness	potential for cost-saving when replacing multiple single-analyte tests	high initial setup costs and maintenance make MS less accessible for smaller clinical laboratories.
clinical utility validation	enables retrospective and prospective cohort studies using archived samples	requires large, diverse patient cohorts to demonstrate clinical utility and generalizability
integration into clinical workflow	MS-based diagnostics can be integrated with electronic health records and lab systems	requires bioinformatics infrastructure and trained personnel not always available in clinics

## 9 Bioinformatic Tools and AI

Data integration and bioinformatics are crucial in translating MS proteomic data into meaningful biological insights. By integration of protein concentration data with external biological knowledge, such as gene ontologies, interaction databases, and pathway maps, it is possible to draw comprehensive panels about cellular mechanisms, disease states, and drug effects.

### 9.1 PPI Networks

PPI networks map the reciprocal relationships between proteins based on experimental data (e.g., co-immunoprecipitation, yeast two-hybrid assays) or computational predictions.^257^ The study of PPIs in the context of proteomics involves mapping out these interactions, visualizing the resulting networks, and performing computational analyses to identify key proteins or clusters of proteins involved in specific biological functions or diseases.^258,259^ A variety of curated databases and repositories provide experimentally validated or computationally predicted PPIs.^260^ One of the most widely used is STRING (Search Tool for the Retrieval of Interacting Genes/Proteins), which aggregates known and predicted PPIs from multiple sources, including experimental data, computational prediction methods, and public text-mining efforts.^261^ STRING assigns a confidence score to each interaction, allowing users to filter interactions based on the strength of evidence.^262^ A conceptual representation of STRING output is reported in Figure 16A. Other popular PPI databases include BioGRID (Biological General Repository for Interaction Data sets), which focuses on experimental data from high-throughput PPI studies, and IntAct, a database that provides detailed information about experimentally validated PPIs, including the methods used to determine the interactions and the organisms in which they were observed.^263–265^ MINT (Molecular INTeraction Database) is another resource that specializes in experimentally determined interactions, with a focus on high-quality, curated interaction data.^265^ In a PPI network, proteins are represented as nodes, while their interactions are represented as edges connecting these nodes.^257^ The resulting networks can vary in complexity depending on the number of proteins identified and the degree of connectivity between them. In large-scale proteomics studies, where thousands of proteins may be identified, networks can become highly complex.^266^ To manage this complexity, computational tools often apply filters to reduce the number of interactions by considering only high-confidence or experimentally validated PPIs.^267^ Network construction is further enhanced by integrating additional data, such as gene expression levels, posttranslational modifications, or protein localization information.^85^ This allows for the generation of context-specific networks that reflect the state of the proteome under particular experimental conditions, such as disease states or drug treatments.^268^

**Figure 16:**
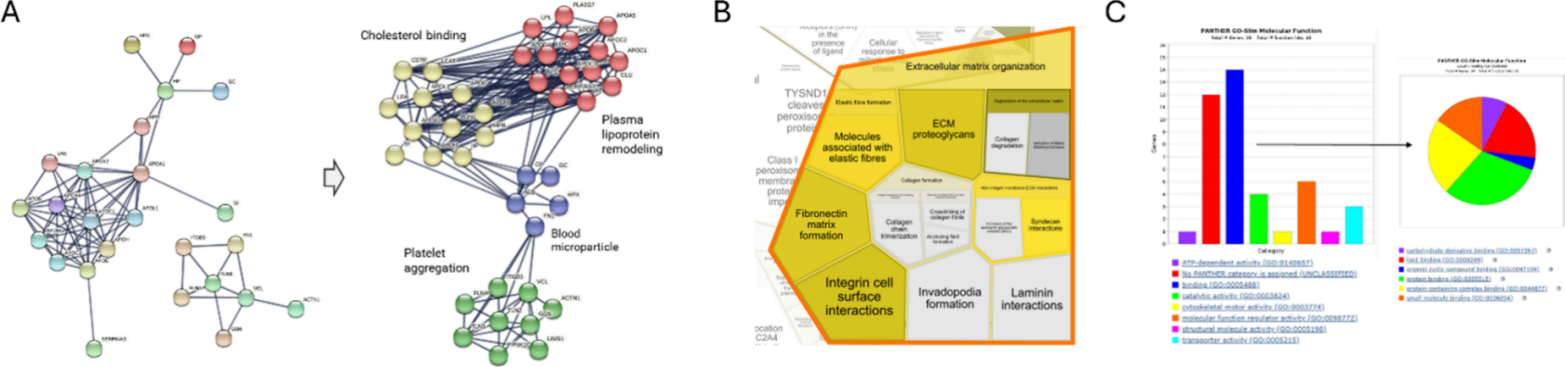
Some of the way to represent bioinformatics data from MS proteomics outcomes. (A) STRING nonenriched network generated with STRING vs its corresponding enriched network (right). (B) Voronoi plot generated with Reactome enrichment tool. (C) Histogram map obtained with GO-Panther encompassing the main biochemical networks identified in an MS proteomics experiment as functional GO (FC), with a further subclass representation (right).

One of the fundamental properties of PPI networks is degree centrality, which measures the number of connections a protein has within the network.^269^ Proteins with a high degree of centrality, often referred to as “hubs,” are typically key regulators or scaffolding proteins that play major roles in organizing protein complexes or signaling pathways.^269^ Hubs tend to be evolutionarily conserved and are often critical for maintaining cellular homeostasis. In disease contexts, hub proteins are frequently dysregulated, making them attractive targets for therapeutic intervention.^270^ Another important topological measure is “betweenness centrality”, which quantifies how often a protein lies on the shortest path between other proteins in the network.^271^ Proteins with high betweenness centrality act as bottlenecks or bridges in communication between different parts of the network, making them important for coordinating diverse cellular functions.^272^ In addition to centrality measures, clustering coefficient is used to assess the degree to which proteins in a network tend to form clusters or modules.^273^ Proteins that interact with each other frequently tend to form tightly connected subnetworks, which can represent protein complexes or functional modules involved in specific biological processes, such as DNA replication, cell division, or metabolic regulation.^274^ By identifying these clusters, it is possible to gain insights into the functional organization of the proteome and identify key regulatory modules that may be perturbed in disease conditions.^268^ Taking into account the practical steps to build an experimental analysis, the selection of proteins to build a PPI network and perform an enrichment analysis requires a combination of rigorous data processing and statistical criteria to ensure the biological validity of the results.^275^ The workflow begins with data preprocessing, which includes the removal of contaminants, reverse and decoy sequences, and proteins with insufficient peptide evidence.^259^ Quantitative data are normalized to correct for technical variation between samples, for instance, using median or total ion current normalization for label-free quantification, or reference channel normalization for TMT-based workflows.^276^ Missing values are imputed according to the assumed mechanism of missingness: missing not at random (MNAR) values are typically imputed with a down-shifted normal distribution to simulate low-abundance features, whereas missing completely at random (MCAR) values may be replaced using nearest-neighbor or Bayesian methods.^277,278^ To ensure data reliability, proteins supported by at least two unique peptides and detected in a minimum of 50 to 70% of biological replicates in at least one condition are typically retained.^279^ Minimum coverage (usually set to 5% or above) can also be used as a filtering parameter.^23^ Once the data set is quality-filtered, statistical analyses are applied to identify proteins that differ significantly between experimental groups. Moderated *t* tests or linear models are commonly used, as they account for technical and biological variance across replicates.^280^ *p*-values are adjusted for multiple testing using the Benjamini-Hochberg procedure to control the false discovery rate (FDR).^281^ Proteins are then selected for PPI and enrichment analysis according to both their statistical significance and magnitude of change.^282^ A widely adopted threshold is an adjusted q-value <0.05 combined with an absolute log_2_ of the normalized abundance ratios (|log_2_FC|) ≥ 0.585 (corresponding to a 1.5-fold overexpression, or 0.5-fold downregulation vs control), though stricter cutoffs (|log_2_FC| ≥ 1.0) may be used for more conservative analyses, sometimes combined with q-value <0.01 in the case of limited biological replicates.^283,284^ Following selection, proteins are annotated with standardized identifiers such as UniProt accessions or official gene symbols to ensure compatibility with databases used for PPI network construction (e.g., STRING) and pathway enrichment.^285^ The background population for enrichment tests should correspond to the set of all proteins reliably quantified in the experiment, rather than the entire theoretical proteome, to avoid enrichment bias due to MS detectability.^286^ In cases in which the list of significant proteins is very large, further refinement can be achieved by prioritizing those with the highest statistical significance or those belonging to pathways of specific biological interest. This can be done, for instance, by ranking proteins by combined significance metrics, such as – log_10_(q-value), or focusing only on the top n proteins ranked by statistical or biological relevance.^287^

### 9.2 Gene Ontology (GO) and Pathway Enrichment Analysis

A major bioinformatic tool used to understand the functional implications of proteomics data is Gene Ontology (GO). GO terms describe proteins in terms of three categories: i. Molecular function (MF), that describe the biochemical activities of proteins (e.g., kinase activity, DNA binding). ii. Biological process (BP), which clusters the larger biological processes in which proteins are involved (e.g., cell division, apoptosis). Cellular component (CC), i.e., where proteins are located within the cell (e.g., nucleus, cytoplasm).^288^ By statistically over-representing certain GO terms, it is possible to deduce which molecular functions or biological processes are significantly enriched in the data set (Figures 16B,C).^17^ Tools like DAVID (Database for Annotation, Visualization, and Integrated Discovery), PANTHER, and GOrilla facilitate this type of analysis by linking the identified proteins with their corresponding GO annotations.^289–292^ In addition to GO analysis, pathway enrichment analysis is used to identify metabolic or signaling pathways that are over-represented in the proteomic data. Tools like KEGG (Kyoto Encyclopaedia of Genes and Genomes), Reactome, and Ingenuity Pathway Analysis (IPA) are commonly employed to link proteins with known biological pathways.^289,293–296^ For example, a study of cancer samples may reveal enrichment in pathways related to cell cycle regulation, DNA repair, or apoptotic signaling. Enrichment analysis is often conducted using statistical tests such as Fisher’s exact test or hypergeometric distribution to determine the significance of pathway over-representation.^292^ These analyses can reveal key signaling pathways that are dysregulated in diseases or altered in response to drug treatment, providing insights into the molecular underpinnings of the biological condition. Advantages and limitations of bioinformatic data analysis of the MS raw data output are reported in Table 9.

**Table 9:** Advantages and Limitations of Bioinformatic Data Analysis of the MS Raw Data Output

advantages	limitations
high-throughput analysis: can identify and quantify thousands of proteins in a single experiment	missing values are common, especially for low-abundance proteins, complicating downstream statistical analysis
quantitative comparisons: allows differential expression analysis across multiple conditions/groups	data normalization and imputation choices significantly influence results
pathway and network analysis: integrates protein lists into biological context (e.g., GO, KEGG, Reactome)	functional annotation is sometimes incomplete or outdated for many proteins, especially nonmodel organisms
statistical rigor: supports significance testing (e.g., q-values, fold change) for robust conclusions	thresholding decisions should be decided (e.g., FC cutoffs, q-value) and the choice affect sensitivity and specificity
automation and reproducibility: same workflows can be applied to different experimental conditions (e.g., Perseus, MSstats, Proteome Discoverer)	tool selection variability: different pipelines may yield different results for the same data set
cross-omics integration: enables combination with transcriptomics, metabolomics, etc for multiomics purposes.	data integration challenges due to discordance between mRNA and protein levels
public databases: access to large reference data sets (e.g., PRIDE, PeptideAtlas, UniProt)	batch effects and technical variability can affect biological signals

### 9.3 AI in Managing MS Proteomics Data

The integration of AI into MS proteomics is still at its beginning. It significantly enhances the analysis and management of complex biological data, marking a transformative shift in the field. AI applications are particularly valuable in handling the high-dimensional data sets typical of proteomics, where traditional analytical methods often struggle.^297^ For instance, deep learning models such as Prosit have been developed to predict fragmentation patterns in MS data, enabling investigators to achieve more accurate protein identifications, especially in highly multiplexed MS/MS spectra typical of DIA, by implementing classical database searches with intensity-based scores derived from AI predictions.^298,299^ Another AI application in MS Proteomics is represented by the Chimerys peptide browser (Thermo Fisher) on the Proteome Discoverer platform. Chimeric spectra often arise when multiple peptides are co-isolated and fragmented in the ms/ms scans. Chimerys has been shown to double the number of peptide identifications compared to classical search algorithms like Sequest HT, achieving identification rates exceeding 80%.^300^ Additionally, it increases the average number of identified peptides per protein by approximately 2.5-fold, translating to an average of two PSMs per spectrum.^301^ Moreover, AI excels in noise reduction and pattern recognition within complex spectra, which is crucial for identifying low-abundance proteins that might otherwise be overlooked.^297^ Machine learning algorithms can effectively denoise spectra, leading to enhanced accuracy in peptide and protein identification.^302^ Predictive modeling is another significant application where AI can anticipate protein behavior based on historical data, thus providing insights into how proteins might respond to specific perturbations or treatments.^303^ This capability extends to predicting protein–protein interactions and posttranslational modifications, which are essential for understanding cellular functions and disease mechanisms.^21^ AI’s role is not limited to data processing. Indeed, it also enhances biological interpretation by linking identified proteins with known biological processes and pathways.^304^ Furthermore, AI facilitates the integration of proteomics data with other omics data (such as genomics and metabolomics), providing a comprehensive view of cellular processes and enabling more robust biological insights.^305^

In addition to these analytical capabilities, AI supports automated hypothesis generation by suggesting potential follow-up experiments based on identified patterns in the data.^306^ For example, if certain proteins are consistently coregulated across different conditions, AI can prompt researchers to explore their functional relationships further.^307^ The development of dynamic visualization tools powered by AI also aids researchers in interpreting complex data sets by highlighting significant areas of interest. Real-time analysis is another promising application of AI in proteomics.^308^ As MS techniques evolve and data generation accelerates, the need for immediate feedback becomes crucial. AI can provide real-time insights during experiments, particularly in live-cell proteomics, where a timely analysis can influence experimental outcomes.

## 10 Integration of MS Proteomics with Other *Omics* Platforms—the Dream

The integration of MS proteomics data with other *omics* techniques holds the ability to accelerate the understanding of complex biological systems.^309^ MS proteomics provides deep insights into the proteome, revealing relative protein abundances and their overall functions in relation to other proteins.^310^ By integration of MS proteomics with other *omics* layers, such as genomics, transcriptomics, metabolomics, and epigenomics, investigators can develop a more holistic view of cellular functions and disease mechanisms. This approach is often referred to as “dual omics” when involving two omics disciplines, or “multiomics” for more, as represented in Figure 17.^311^

**Figure 17:**
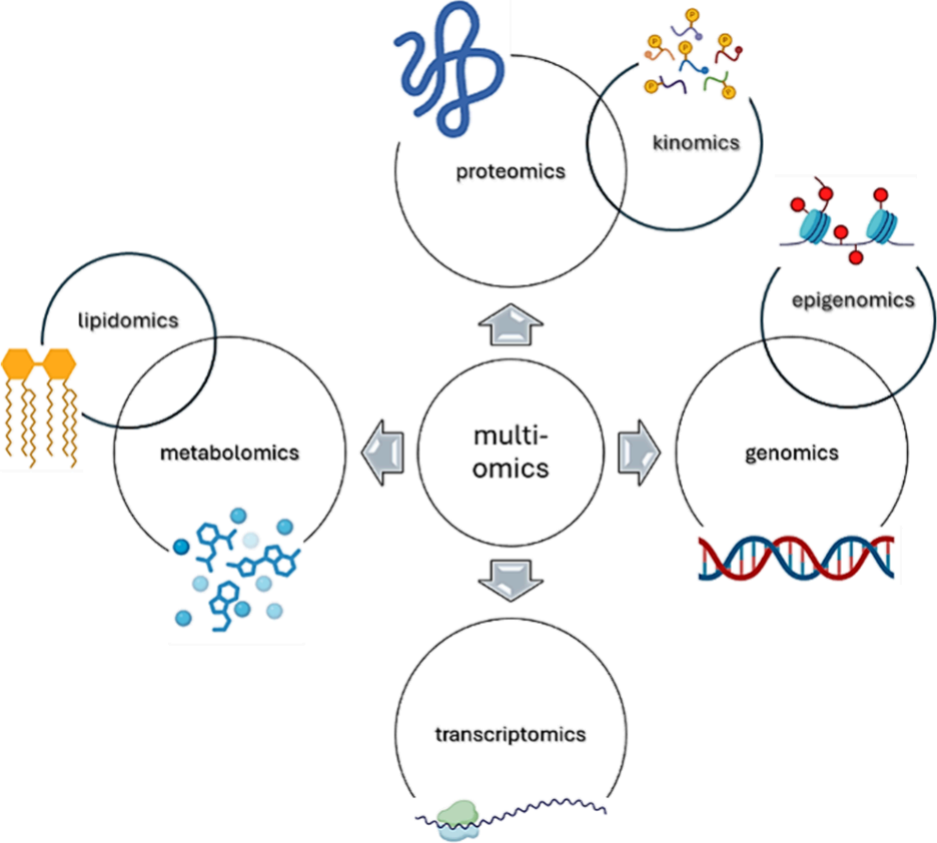
Depiction of the multiomics MS-based panorama, splitting in MS proteomics, genomics, transcriptomics, and metabolomics, and their respective subtechniques. Indeed, MS phosphoproteomics, or kinomics, is a phosphate-focused MS proteomics; epigenomics is the study of DNA methylation and other regulation mechanisms, and lipidomics is a form of MS metabolomics experiment focused on biological analytes with a lipid nature.

Genomics, which provides information about an organism’s entire DNA sequence and gene variants, lays the basis for understanding the potential of biological systems. However, not all genetic variations lead to observable phenotypic changes, and gene expression does not always correlate with protein levels due to posttranscriptional and posttranslational modifications. By combining MS proteomics and genomics, it is possible to fill the gap between the genetic code and protein function.^312^ This integration, often referred to as proteogenomic, is particularly useful for identifying novel protein-coding regions, alternative splicing events, and the effects of mutations on protein function in diseases such as cancer.^312^ Transcriptomics, which analyses RNA expression levels, complements MS proteomics by showing which genes are actively being transcribed.^313^ Yet, mRNA levels do not always correlate with protein abundance due to translational regulation and protein degradation. Integrating proteomics with transcriptomics enables a better understanding of the layers of gene regulation, revealing how variations in RNA expression translate into functional proteins.^314^

Metabolomics, the study of small molecules and metabolites within a biological system, offers another layer of valuable information.^315^ Proteins, as enzymes, drive metabolic reactions, and the metabolome reflects the biochemical activity occurring in cells and tissues.^316^ By integrating MS proteomics with metabolomics, it is possible to connect protein expression levels and modifications to specific metabolic pathways, revealing how protein function impacts metabolic fluxes. This approach is essential in understanding disease metabolism, drug responses, and metabolic disorders.^317^ Epigenomics, the study of chemical modifications on DNA and histones that affect gene expression, can also be integrated within the MS proteomics pipeline.^318^ Epigenetic modifications influence chromatin structure and accessibility, which in turn affects which genes are transcribed into RNA and ultimately translated into proteins.^3^ The integration of epigenomics and proteomics is helpful for studying how epigenetic changes alter protein expression in multifactorial diseases like cancer and neurological disorders.^319^ After preprocessing and quality control assessment, the core of a multiomics workflow lies in the analytical strategies that allow data integration across distinct molecular layers. Since each omics data set (genomics, transcriptomics, proteomics, metabolomics, epigenomics, etc.) has unique characteristics in terms of dimensionality, measurement error, dynamic range, and biological interpretation, the design of the analysis pipeline strongly influences the type of insights that can be drawn. Numerous platforms were also designed to analyze in parallel MS Proteomics, RNA-seq data, and metabolomics data and combine the output to a single, unified multiomics biochemical network. These platforms include mixOmics (an R platform), which can deal with all the three techniques and uses multivariate analysis, MOFA (multi-omics factor analysis), iClusterPlus, and SNF (Similarity Network Fusion), ideal for cluster analysis, and others.^320–323^ More user-friendly ecosystems, mostly web-based, include PaintOmics and Perseus, which are mainly proteomic-centered, but when provided with already analyzed RNA-seq or metabolomics data from other platforms, are able to merge the information and build correlation plots as well as integrated KEGG and GO pathway levels.^324,325^ The integration of different omics layers is possible thanks to the creation of integrated databases like CPTAC, a compendium of a compendium of tissue samples from different cancer types, along with their respective healthy tissue samples.^326^ Multiomics data analysis approaches are usually classified into three broad categories: early integration, intermediate integration, and late integration, that in practice are often combined or tailored depending on the biological question and the data available.^327,328^ Early integration refers to approaches in which features from different omics layers are merged into a single data matrix before statistical or ML analyses. This strategy treats all omics features as comparable variables, often after normalization or transformation to bring them onto a common scale.^329^ The simplest examples are concatenation-based methods, where transcript counts, protein abundances, and metabolite intensities are stacked together, and dimensionality reduction techniques such as PCA or clustering algorithms are applied to the combined data set. This is the case of MONIER, a novel multiomics early Integration framework addressed to disease diagnosis and biomarker discovery.^330^ Early integration has the advantage of simplicity and provides a global view of the molecular system, to allow unsupervised methods to detect overarching patterns, patient subgroups, or molecular signatures that span multiple omics levels.^331^ However, its substantial challenges are the differences in feature numbers (e.g., tens of thousands of transcripts versus a few hundred metabolites), and the missing values can bias analyses toward the most abundant or best-measured data type.^332^ In addition, early integration does not explicitly model the hierarchical or causal relationships between omics layers, which may limit interpretability.^333^ Intermediate integration methods attempt to overcome these limitations by jointly modeling omics data sets while preserving the structure and uniqueness of each layer. Instead of concatenating all features, these methods extract latent variables or factors that capture shared variance across data sets while also retaining data set-specific variation.^334^ Canonical correlation analysis (CCA), as provided in Smccnet 2.0 toolm, is a classic example as it identifies linear combinations of variables that maximize correlations between omics layers.^335^ More recent developments include partial least-squares (PLS)-based frameworks, as implemented in DIABLO (Data Integration Analysis for Biomarker discovery using Latent cOmponents), which enable supervised integration of multiomics data in relation to an outcome variable.^336^ Similarly, MOFA provides an unsupervised Bayesian framework that decomposes variability into factors representing both shared and modality-specific signals.^323^ Other matrix factorization, manifold learning, and deep-learning-based methods (such as variational autoencoders) have also been developed to capture nonlinear relationships between omics types.^337^ The strength of intermediate integration is its ability to balance global structure with omics-specific insights, offering interpretable models that can highlight both cross-omics associations and unique biological contributions from each data set.^338^ Late integration represents the most conservative strategy, in which each omics layer is analyzed separately, and the results are integrated at a higher level of biological interpretation.^339^ This approach is particularly valuable when data sets are heterogeneous in size and/or quality, since it avoids forcing them into a single modeling framework.^339^ Network-based late integration, as in the MOLI method proposed by Sharifi-Noghabi et al., is also common, where omics-specific networks (e.g., coexpression or PPI maps) are generated independently and then overlaid to reveal cross-layer regulatory modules.^340^ The downside is that late integration may miss subtle, multilayer associations that only emerge when data sets are modeled together.^341^ However, because it allows maximum flexibility and makes use of well-established single-omics pipelines, it still remains the most widely used in the scientific literature, especially in translational and clinical studies where robustness of each independent omics experiments and interpretability are prioritized.^333^ In practice, the choice between early, intermediate, and late integration is not absolute but rather depends on the research context and the aims of the investigation. Early integration is often exploratory and well suited for discovering broad patient stratification or molecular subtypes. Intermediate integration offers a powerful balance of depth and interpretability, and it is particularly useful in biomarker discovery or mechanistic studies, where the interplay between omics layers is of interest. Late integration remains valuable when dealing with heterogeneous data sets, legacy data, or when interpretability and reproducibility take precedence.^342^ Increasingly, hybrid strategies are being developed, for instance, combining late integration of pathway analyses with intermediate modeling of molecular networks (as in the directional P-value merging (DPM) method), to capture the complementary strengths of each approach.^85^

In conclusion, the integration of MS-based proteomics with complementary omics disciplines provides a systems-level framework that can accelerate drug discovery by connecting genomic variation, transcriptional regulation, protein dynamics, and metabolic fluxes into unified molecular networks. While challenges related to data dimensionality, missing values, and cross-platform normalization persist, the refinement of early, intermediate, and late integration algorithms, together with the development of robust computational ecosystems, is progressively overcoming these limitations. As these approaches mature, MS proteomics embedded within multiomics integration will be the key for biomarker discovery, early-stage drug discovery, and mechanism-based therapeutic design, ultimately enhancing translational impact in medicinal chemistry and making more fruitful the collaboration between chemists, pharmacologists, and clinicians.

## 11 Perspectives

MS-based proteomics is quickly becoming an indispensable tool in modern drug discovery programs, delivering unparalleled information regarding protein expression, interactions, and posttranslational modifications. MS proteomics can be applied to all stages of the drug development pipeline, from target identification and validation to MoA clarification, biomarker identification, and clinical studies. The hallmark of one of the most significant strengths of MS proteomics lies in its ability to analyze proteins within their native biological environments. This is a possibility with chemoproteomics, e.g., with ABPP, HDX-MS, XL-MS, and other ad hoc experiments, which allow us to investigate protein functions and conformational changes with high sensitivity. These technologies provide a degree of biological information above that of traditional transcriptomic or genomic approaches. Recent technological developments in chemoproteomics methodologies, including TPP-MS and PAL, have significantly improved the capability to study drug–target interactions and off-target activities in complex biological systems. These approaches have been crucial in identifying direct targets of synthetic and natural product drugs, drug resistance mechanisms, and drug repurposing targets. Furthermore, the integration of MS proteomics into FBDD has enabled early-stage development through the capacity to rapidly, label-free confirm fragment binding and the stoichiometry of interactions.

At the preclinical stage, MS-based proteomics plays a central role in characterizing drug candidates’ mechanisms of action, identifying off-target activities, and establishing their toxicity profiles. XL-MS, Co/IP-MS, HDX, LiP-MS, and ABPP enable the exploration of protein interaction with their ligands and with other proteins, providing a systems-level understanding of the drug MoA. Such data are essential for lead optimization and reduction of late-stage attrition. Moreover, MS proteomics also provides the possibility of identifying pharmacodynamic biomarkers that are important for drug efficacy and safety monitoring within clinical trials. By analyzing patient samples in therapeutic regimens at different time-lapses, translational investigators can simplify the evaluation of the molecular effect of therapeutics, thus making adaptive trial designs and personalized treatment regimens possible. As the field advances toward systems biology and individualized medicine, MS proteomics has stood the test of translational research. Clinical specimen analysis, from tissue biopsy to ascitic fluid and plasma, has aided in identifying disease-specific biomarkers and facilitated the stratification of patients. Protein information integration with clinical metadata is opening the gates for more precise, data-informed treatment choices and overcoming bench-to-bedside translational gaps.

Since 2024, single-cell proteomics has represented a rapidly advancing field at the intersection of MS and cellular biology, aimed at characterizing the proteome of individual cells rather than bulk populations. Traditional proteomic workflows average signals across thousands to millions of cells, thereby masking the heterogeneity that underlies critical biological processes such as differentiation, tumor evolution, and drug resistance. By contrast, single-cell proteomics leverages ultrasensitive sample preparation strategies (e.g., nanodroplet-based digestion, acoustic and microfluidic isolation) and state-of-the-art mass spectrometers (Orbitrap Astral from Thermo Fisher and timsTOF SCP from Bruker) to achieve reliable detection of proteins from a single cell. Quantification strategies include both label-free approaches and isobaric labeling with carrier proteome boosting (as pioneered in SCoPE-MS and extended in SCoPE2 and nPOP), which mitigate missing data. However, the field still faces technical and conceptual limitations. Sensitivity remains a major bottleneck, as protein copy numbers span several orders of magnitude, and many low-abundance proteins remain undetectable. Quantification accuracy is challenged by ion undersampling (i.e., still an important amount of signals is not detected by current MS proteomics experiments, due to low abundance), ratio compression in multiplexed analyses, and stochastic sampling in DDA, although DIA and PASEF (Parallel Accumulation Serial Fragmentation, by Bruker) have alleviated some of these issues.

MS proteomics has also allowed the development of the so-called “drug fingerprinting,” the proteome-based analogue to gene expression profiling in transcriptomics (e.g., LINCS project), which captures functional and post-translational responses, i.e. it aims to translate the concept of transcriptomic perturbation profiling (as exemplified by the Connectivity Map, https://www.broadinstitute.org/connectivity-map-cmap) into the proteomic domain. The concept is to associate a chemical scaffold with whole cellular modifications. This technique may be implemented to drive drug discovery within a new, extended concept of SAR, by expanding the activity of the drug from a single target to the whole biochemical modifications induced by the considered chemical scaffold. A landmark example of MS proteomic drug fingerprinting is the ProTargetMiner resource, which systematically profiles the effects of ∼50 kinase inhibitors across multiple cancer cell lines using quantitative MS-based proteomics. This study demonstrated that proteomic responses could classify compounds according to their known mechanisms and uncover unanticipated off-target activities. More recent efforts, such as ProteomeHD, have extended this principle to broader chemical spaces and cellular systems, driving the research toward comprehensive proteome-level connectivity maps analogous to transcriptomic databases.

Furthermore, the exploitation of machine learning (ML) in MS proteomics workflows is a promising frontier. ML-based tools are being utilized more to address the high-dimensional data sets generated by modern mass spectrometers, enhancing sensitivity, reproducibility, and predictive modeling of drug toxicities and response. Programs such as MaxQuant, Skyline, Progenesis and Proteome Discoverer, as well as emerging AI-enabled platforms, have accelerated data interpretation and confidence, further bolstering MS proteomics’ potential for high-throughput drug screening and mechanistic exploration. The integration of MS-based proteomics into drug discovery has led to the generation of enormous and intricate data sets, which call for stable data storage and management solutions. Traditional file-based systems are not able to handle the quantity and complexity of proteomic data, which leads to the retrieval, analysis, and sharing problems of the data. To address these challenges, various efforts have been put in place to standardize data formats and make data sharing easier and quicker. An example is the ProteomeXchange consortium, which provides a common framework for data submission and distribution across several MS proteomics repositories, i.e., PRIDE, PeptideAtlas, and MassIVE. These repositories support data sharing and reanalysis through providing standardized formats and rich metadata annotations, which is also critical in ensuring the reproducibility and reusability of proteomics data. Thorough annotation of experimental conditions, procedures in sample treatment, and analysis parameters must be performed to facilitate meaningful interpretation and comparison of the data. Efforts like the Proteomics Standards Initiative (PSI) have aided in the development of standardized data formats and controlled vocabularies (e.g., the Minimum Information about a proteomics experiment (MIAPE) standard format) to ensure greater levels of metadata consistency. Furthermore, platforms such as iProX have embraced big data technologies in managing the growing size of proteomics data sets. By scalable design and fast data retrieval systems, iProX supports the storage and rapid data retrieval of large quantities of MS proteomic data, facilitating high-throughput analysis and retrospective investigations.

With the increase in MS proteomics workflows, coupling with other omics technologies such as genomics and metabolomics will help to bring a better understanding of disease mechanisms and therapeutic response. The development of advanced bioinformatics tools will again enhance the analysis and interpretation of high-complexity proteomic data and result in more efficient and targeted approaches to drug discovery. Also, its increasing levels of automatization from sample processing to data analysis and the always more consistent miniaturization of high-performance LC–MS/MS systems will allow high-quality data to be obtained and analyzed in large batches. On the other hand, limitations to the full exploitation of this technique still exist and include the consistent costs of MS Proteomics platforms (both as MS machines purchase and their maintenance, including specialized and dedicated personnel and consumables) and of data storage clouds and platforms, e.g., supercomputers. Also, omics sciences are intrinsically affected by interlaboratory reproducibility issues, as currently, few to no operative standard procedures are internationally recognized, especially for the label-free MS proteomics experiment. However, as for nearly any other technological applications and advancements of the most recent era, the costs of HRMS will become more and more affordable. With ongoing collaboration across academia, industry, and regulatory bodies, it is foreseeable that standardized, scalable, and cost-effective MS proteomics workflows will soon become an essential part of the pharmaceutical toolbox to accelerate the drug discovery process associated with biomedical investigations.

## †

Department of Food and Drug Sciences, University of Parma, Parco Area delle Scienze 27/A, 43124 Parma, Italy

## Abbreviations

ABPP: activity-based protein profiling
AOP: adverse outcome pathway
CID: collision-induced dissociation
Co-IP-MS: co-immunoprecipitation followed by MS
DDA: data-dependent acquisition
DEP: differentially expressed protein
DIA: data-independent acquisition
FBDD: fragment-based drug design
FPLC: fast protein liquid chromatography
HCD: higher collision dissociation
HDX-MS: hydrogen–deuterium exchange mass spectrometry
HRMS: high-resolution mass spectrometry
HTS: high-throughput screening
IMAC: immobilized-metal affinity chromatography
LBDD: ligand-based drug design
LFQ: label-free quantification
MD: molecular dynamics
MRM: multiple reaction monitoring
MS: mass spectrometry
PAL: photoaffinity labeling
PIN: protein interaction network
PPI: protein–protein interaction
PRM: parallel reaction monitoring
PTM: posttranslational modification
SBDD: structure-based drug design
SRM: single reaction monitoring
TIC: total ion current
TMT: tandem mass tag
TPP-MS: thermal proteome profiling mass spectrometry
WB: Western blot
XIC: extracted ion chromatogram
XL-MS: cross-linking mass spectrometry
